# Designing optimal integrated electricity supply configurations for renewable hydrogen generation in Australia

**DOI:** 10.1016/j.isci.2021.102539

**Published:** 2021-05-15

**Authors:** Muhammad Haider Ali Khan, Rahman Daiyan, Zhaojun Han, Martin Hablutzel, Nawshad Haque, Rose Amal, Iain MacGill

**Affiliations:** 1 Particles and Catalysis Research Laboratory, School of Chemical Engineering, The University of New South Wales, Sydney, NSW 2052, Australia; 2 Siemens Limited, Melbourne, VIC 3153, Australia; 3 CSIRO Energy, Private Bag 10, Clayton, VIC 3169, Australia; 4 Collaboration on Energy and Environmental Markets, The University of New South Wales, Sydney, NSW 2052, Australia

**Keywords:** Electrochemistry, energy policy, energy engineering, energy sustainability, energy flexibility, energy systems

## Abstract

The high variability and intermittency of wind and solar farms raise questions of how to operate electrolyzers reliably, economically, and sustainably using predominantly or exclusively variable renewables. To address these questions, we develop a comprehensive cost framework that extends to include factors such as performance degradation, efficiency, financing rates, and indirect costs to assess the economics of 10 MW scale alkaline and proton-exchange membrane electrolyzers to generate hydrogen. Our scenario analysis explores a range of operational configurations, considering (i) current and projected wholesale electricity market data from the Australian National Electricity Market, (ii) existing solar/wind farm generation curves, and (iii) electrolyzer capital costs/performance to determine costs of H_2_ production in the near (2020–2040) and long term (2030–2050). Furthermore, we analyze dedicated off-grid integrated electrolyzer plants as an alternate operating scenario, suggesting oversizing renewable nameplate capacity with respect to the electrolyzer to enhance operational capacity factors and achieving more economical electrolyzer operation.

## Highlights

• A comprehensive framework to assess levelized cost of hydrogen (LC_H2_) is developed.
• Current and future outlook of LC_H2_ for the electrolyzer operations in Australia.
• LC_H2_ ≤ A$3 kg^−1^ requires electrolyzer cost < A$500 kW^−1^, < A$30MWh^−1^, and WACC of <3.
• Oversizing dedicated solar and wind capacity by 1.5 times led to 10% decrease in LC_H2._

## Graphical abstract

**Figure:**
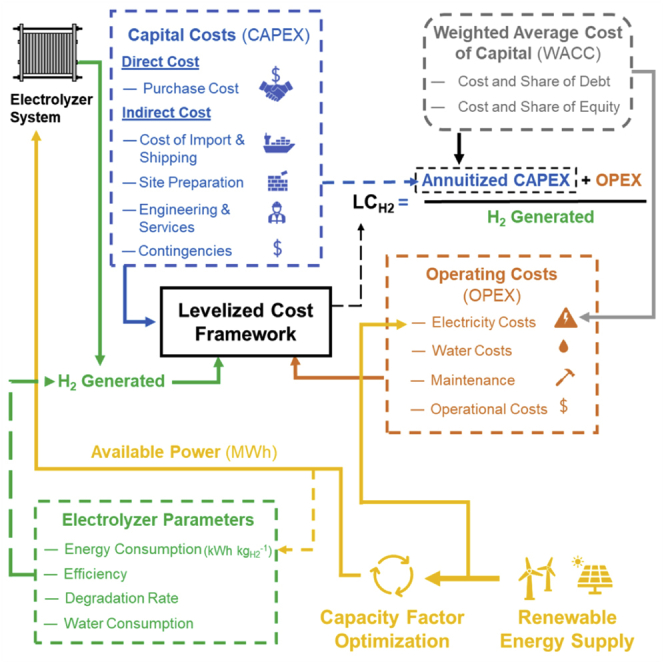

## Introduction

The world's continued reliance on fossil fuels is leading to declining reserves, which at some point can threaten energy security while also contributing to global climate change (Mason, 2003; Krane, 2017). Several renewable generation options show great promise in improving long-term energy security and reducing emissions from the electricity sector. Wind- and solar-based electricity generation, in particular, has seen rapidly declining costs over the past decade and is now a highly cost-competitive new generation option (IRENA, 2020b). However, these technologies exhibit highly variable and somewhat unpredictable outputs on daily to seasonal timescales. Thus, intermediate storage through the development of new flexible and dynamic industrial electricity loads like power-to-gas (PtG) can play a key role in facilitating greater wind and solar deployment, enabling a more sustainable and reliable energy supply(Blanco and Faaij, 2018). Moreover, deployment of PtG plants can also help address fossil-fuel-dominated and hard to address non-electricity energy sector emissions by converting renewable electricity into industrial feedstocks, e.g., methane or hydrogen (H_2_), which can then be used as a low carbon downstream alternative (Lewandowska-Bernat and Desideri, 2017). Among PtG pathways, H_2_ generation is especially significant (Götz et al., 2016), as renewable H_2_ can then be readily used downstream as a clean alternative energy carrier across the energy sector in transportation and domestic, commercial, and industrial energy applications (Staffell et al., 2019). Given this potential, renewable hydrogen is increasingly expected to play a vital role in the future sustainable energy market. While the extent of this role is still unclear, some projections see the demand for green hydrogen projected to reach 300 million tonnes by 2070 (IEA, 2020).

Achieving production of this scale will be a transformation. At present, most commercial H_2_ production worldwide is either done through steam methane reforming or coal gasification (Haryanto et al., 2005). Given the environmental impacts of coal and gas utilization and their ultimately limited resource availability, renewable H_2_ production from water electrolysis can thus very well emerge as the favored process over time, especially if effective climate change action is to be undertaken (Bhandari et al., 2014). Nevertheless, such a transition to renewable hydrogen hinges on developing viable configurations and operating strategies for electrolyzers within electricity markets to take advantage of low-cost but highly variable renewable electricity. Development of such configurations will require a more in-depth and robust understanding of both costs of deploying electrolyzers and their integration into electricity markets with high renewable penetrations.

Prior works in this space have developed frameworks for evaluating the levelized costs of generating hydrogen (LC_H2_) through different electrolysis technologies around the world. These include comprehensive frameworks such as the “H2A” cost model developed by the US National Energy Research Laboratory (NREL) (Steward et al., 2012) and the model suggested by Bertuciolli et al. for the EU's Fuel Cell and Hydrogen Joint Undertaking (FCHJU) (Bertuccioli et al., 2014). However, most recent studies have mainly focused on analyzing single electricity supply configuration (grid powered or either solar or wind) (Ayodele and Munda, 2019; Jiang et al., 2019; Nguyen et al., 2019) or single electrolyzer technology (alkaline electrolyzer [AE] or proton-exchange membrane electrolyzer [PEM]) (Lee et al., 2017; de Fátima Palhares et al., 2018), by either analyzing the economics of proposed hypothetical electrolyzer designs (Vincent et al., 2017; Vincent and Bessarabov, 2018; Taner et al., 2019) or optimizing the electrolyzer for a specific application with assumed capacity factors (Abdin and Mérida, 2019; Kong et al., 2020; Micena et al., 2020). For example, recently, Guerra et al. applied the hydrogen cost models developed by NREL to evaluate the LC_H2_ in the US using grid-supplied electricity using PEM (Guerra et al., 2019). The International Energy Agency (IEA) and International Renewable Energy Agency (IRENA) have analyzed grid and renewable-powered electrolysis configurations, albeit their works do not consider indirect costs, efficiency changes, and technological differences (like stack lifetimes, capital costs, etc.) between AE and PEM systems in affecting LC_H2_ (IEA, 2019; IRENA, 2019, 2020a). Similarly, Shaner et al. considered indirect costs to develop a thorough model for solar photovoltaic (PV)-coupled electrolysis, albeit it fell short of considering tax rates and degradation and considered a simplistic view on stack lifetimes and replacements (Shaner et al., 2016). Even in our recent analysis, where we modeled LC_H2_ using Monte-Carlo-based uncertainty analysis, we did not consider indirect costs and limited our analysis to only a stand-alone PV electrolyzer system (Yates et al., 2020). Nicita et al. also carried out a financial analysis of solar-powered electrolysis to assess the feasibility of hydrogen generation and the potential for selling the by-product oxygen (Nicita et al., 2020). While their model included a detailed consideration of capital costs (direct + indirect costs) and operating costs, it analyzed systems at a smaller scale and did not consider capacity factor optimization or cost trends. Gallardo et al. also recently investigated a solar-driven electrolysis system as a stand-alone or grid-supplied operation, but they too limited their focus on AE systems (Gallardo et al., 2020). Even analysis that focus on comparing AE and PEM electrolyzer costs like the ones conducted recently by the Longden et al.(Longden et al., 2020) and Bruce et al. (Bruce et al., 2018) as part of the Australian National Hydrogen Roadmap, consider optimistic electrolyzer costs, and do not expose how differences in AE and PEM parameters (stack lifetimes, efficiencies etc.) will affect LC_H2_. From such literature reviews, it is clear that there is a need for an inclusive framework that considers key electrolyzer parameters, i.e., cost aspects like direct and indirect capital costs, performance parameters of different stack lifetimes, degradation rates, operating loads, etc., to assess cost of generating hydrogen from both AE and PEM technology under different electricity supply configurations in the near and long term.

Our work aims to address these gaps by developing such an integrated techno-economic assessment tool and demonstrating its use to assess the case of green H_2_ production in Australia. Australia's National Electricity Market (NEM) presents a useful case study as it is exhibiting growing penetrations of low-cost wind and solar generation and great potential for further deployment. Moreover, there is the availability of significant areas across the country that offer excellent wind and solar resources and access to infrastructure (especially natural gas grids to transport H_2_), which can be converted to major new hydrogen hubs despite being disconnected from the national grid (Feitz et al., 2019). These advantages are well recognized by the Australian government and industry, driving growing policy and investment to utilize Australia's renewable potential to generate hydrogen both for domestic and export opportunities (Bruce et al., 2018). Several industrial projects are already under development that include mega projects like the Asia Renewable Energy Hub (15 GW) and Murchison Renewable Energy Hub (5GW) that are expected to be among the largest green hydrogen facilities once operational (Parkinsona, 2019; Matich, 2020). To realize this opportunity and position the country as a large-scale producer and exporter of H_2_ to Asia and beyond, the Australian government has set a target hydrogen production price at ~ A$2 kg^−1^ (Australian Government, 2020). However, the pathway to achieving these cost targets still remains uncertain, making our analysis framework and associated tool to design and evaluate renewable hydrogen electrolysis projects highly industry and policy relevant. In the study presented in this paper, we estimate the present and future levelized cost of producing renewable H_2_ (LC_H2_) under various possible present and future scenarios. Our study extends beyond the existing state of the art with the following contributions:

A techno-economic and financial costing model is developed for commercially available 10MW electrolyzers—both alkaline (AE) and PEM —including electrolyzer performance degradation, financing schemes, and other factors relevant to commercial investment decision-making. This framework extends beyond the existing modeling work which does not consider these parameters in depth (as discussed above). We reveal that these parameters can change LC_H2_ by up to 30%, highlighting the significance of considering these factors in estimating hydrogen production costs.
Explored potential renewable electricity supply configurations in wholesale electricity provision within the NEM to drive electrolyzers with particular focus on power purchase agreements (PPAs) with grid-connected or dedicated utility wind and solar projects to secure low cost, if rather variable electricity supply over the long term.
In particular, we highlight the tradeoffs between achieved capacity factors and costs of electricity provisions with a high share of variable renewable energy, H_2_ electrolyzer proponents (capital/operating costs and performance), and the eventual LC_H2_.
We present a simple yet useful method for assessing the potential tradeoff of oversizing the wind and solar projects (both PPAs and dedicated supply) to improve electrolyzer capacity factor, albeit at the expense of increased capital and operating costs of the renewable generator. We find that oversizing dedicated solar PV or wind farm capacity by 1.5 times causes the LC_H2_ to decrease by up to 10%.
Comparison of LC_H2_ of long-term supply projects (20 years) under different investment windows: window 1 (constructed: 2020) with current electrolyzer costs and performance parameters and window 2 (constructed: 2030) considering the forecasted decline in the electrolyzer capital costs, improvement in electrolyzer performance, and renewable electricity pricing over the decade.
We suggest an ideal mix of electrolyzer capital costs, electricity pricing, and financing schemes to achieve the Australian government's A$2 kg_-1_ target.

Our findings highlight that the current cost of hydrogen from stand-alone wind and solar projects (financed over 20 years) utilizing renewable electrolysis in Australia might be in the range of A$4–12 kg^−1^, with the wind-operated systems offering lower end costs. On the other hand, these costs might vary between A$6 and 8 kg^−1^ if the electrolyzers are operated at the current wholesale electricity pricing within the NEM. Moreover, our findings suggest that the cost of generating hydrogen can be reduced to A$2–3 kg^−1^, provided the electrolyzer capital costs of the order of A$700 kW^−1^ are achieved (including indirect costs), and renewable electricity can be sourced at a price < A$30 MWh^−1^ and/or the projects can be financed with the cost of capital below <4%. Though our model is currently Australia specific, it can be replicated for other jurisdictions by updating the cost assumptions and electricity market data.

The rest of this paper is structured as follows. The results and discussion section outlines our developed framework, especially highlighting the considerations in establishing the electrolyzer's capital and operating cost models. We then explore the electricity pricing trends in the Australian NEM and establish potential electricity supply options to operate the electrolyzers that include (i) high-capacity factor grid-based operated electrolysis, (ii) intermittent renewable PPA-operated electrolyzers, and (iii) optimized dedicated renewable operated electrolyzers. We further build toward providing an in-depth comparative analysis of LC_H2_ for these different supply configurations. Though our particular focus is on renewable electrolysis, we firstly present the case of grid-based electrolysis as a benchmark of high capacity electrolyzer operation and compare it with the exclusively renewable (PPA and dedicated supply) driven electrolysis. In the end, we summarize the key advantages and disadvantages of each operational configuration and their implication on green hydrogen generation in Australia. The experimental procedure section highlights the key assumptions and parameters involved in designing the framework. Supplemental information has also been provided to support our findings.

## Results

### Framework

**Figure 1:**
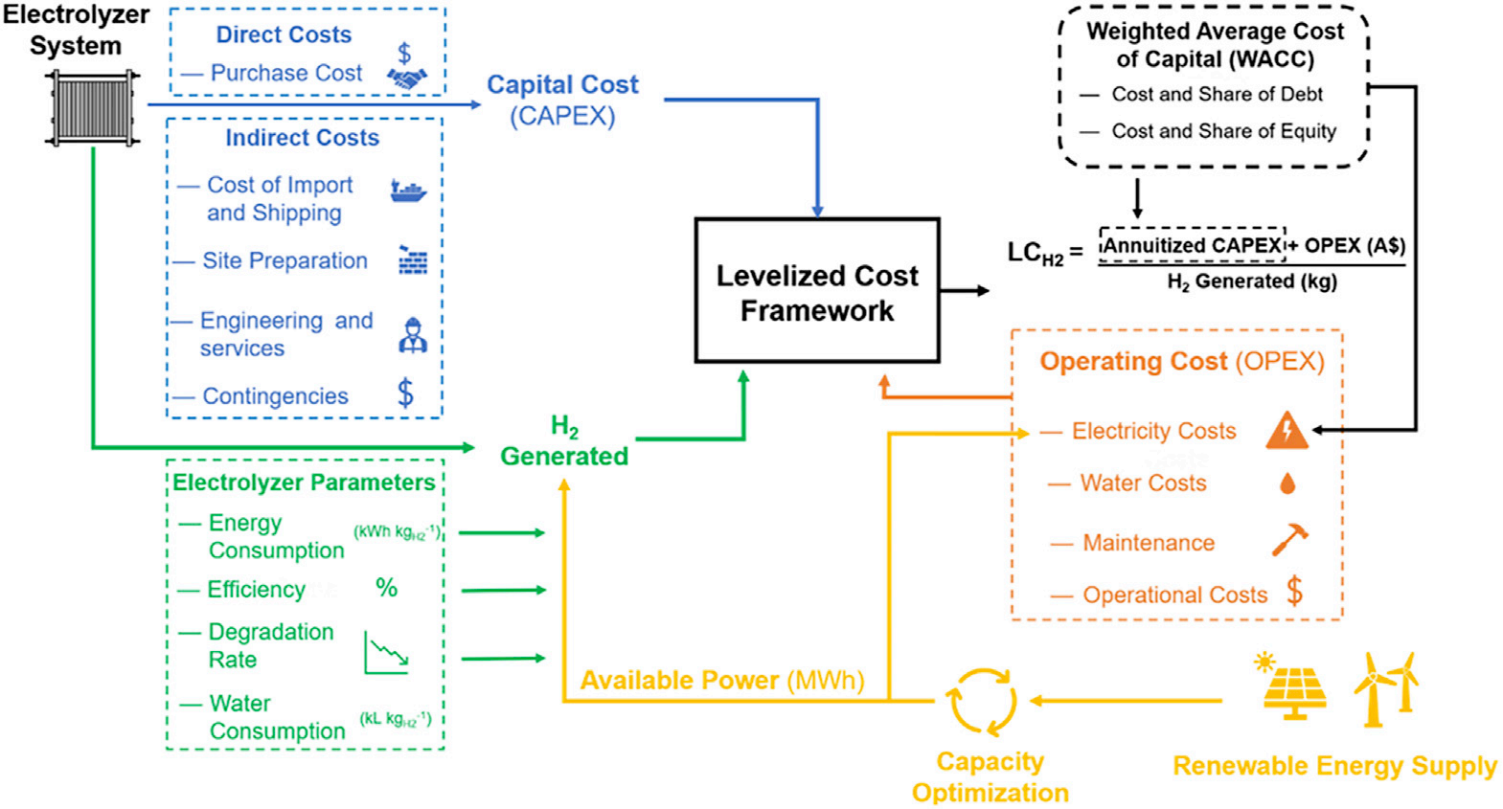
Simplistic representation of analysis framework The flow diagram shows the major technical and economic parameters that influence the levelized costs and their correlation.

Figure 1 represents the underlying framework developed in this work, which builds on the gaps identified in the previous analysis (Table S1). A key focus of the framework is the electrolyzer system (Figure S1) and establishing the system's capital cost. This capital cost includes the direct costs incurred while purchasing the electrolyzer (retail cost) and the indirect costs that would incorporate the costs associated with importing, transporting, and installing the plant in Australia (*vide infra*). We also focus on the performance of the electrolyzer parameters such as specific energy consumption (SEC) and water requirement to determine the feedstock consumption or variable operating costs. Moreover, we also consider that the electrolyzer would require periodic maintenance and will undergo major overhauling during stack replacement, adding to fixed operating costs. Besides, we also consider factors including inflation, taxation, depreciation, and project financing (represented as miscellaneous costs). We also consider the electrolyzer performance factors such as efficiency and stack degradation as they play a role in establishing the amount of hydrogen we generate. To model these costs and parameters, we took data from the commercial electrolyzer companies and literature (refer to STAR Methods – Tables S2–S5).

The other focus of the work is establishing optimal electricity configurations, as this will drive the electricity price (major operating costs) and define the electrolyzer operating levels (capacity factor). To model the electricity supply configurations, we consider data for the Australian NEM provided by the Australian Energy Market Operator (AEMO) (see STAR Methods). The NEM price data were used to determine the cost of electricity supply (Equation 1) which is an essential component of the levelized cost of hydrogen—LC_H2_ (Equation 2). We also analyze the influence of project financing schemes on the required return on capital investment (Equation 3) and the LC_H2_, by considering different weighted average cost of capital (WACC – Equation 4), refer to STAR Methods. These cost and electrolyzer/electricity market performance parameters used in the study are summarized in Table 1.

**Table 1:** Electrolyzer performance, capital, and operating cost parameters.

Parameter	Value	Comment
**Electrolyzer Parameters:**		
Specific energy consumption	AE: 48 kWh kg_H2_^-1^ (2020) to 43 kWh kg_H2_^-1^ (2030) PEM: 54 kWh kg_H2_^−1^ (2020) to 45 kWh kg_H2_^1^ (2030)	The parameters considered are detailed further in the STAR Methods (Table S6)
Water consumption	11 L kg_H2_^−1^ (AE) to 10 L kg_H2_^−1^ (PEM)	
Nominal output range	10–100% of rated capacity	
Degradation rate	Max: 1% loss in output per year	
Stack life	40,000 hr (AE) 40,000 hr (PEM)	
**Capital Expenditures—CAPEX:**		
*Direct CAPEX cost*		
AE system PEM system	2020: Avg.: A$1,353 kW^−1^ (A$594–2,516 kW^−1^) 2030: Avg.: A$1,060 kW^−1^ ($540–1,548 kW^−1^) 2020: Avg.: A$1,803 kW^−1^ (A$1,124–2,218 kW^−1^) 2030: Avg.: A$1,060 kW^−1^ (A$401–2,039 kW^−1^)	The direct cost represents the actual upfront uninstalled cost of the electrolyzer system (stack + balance of plant [BoP]). The avgerage value represents the average costs, which is the mean of the values in brackets that represent the possible range. (Refer to STAR Methods)
*Indirect CAPEX cost*		
Electrolyzer import cost	15% (2020) to 10% (2030) of direct cost	Breakdown of the indirect costs is detailed in STAR Methods
Construction and site preparation cost	19% of direct cost (9% labor and 10% material)	
Engineering service and licensing cost	1% of direct cost	
Project contingency	15% (2020) to 10% (2030) of direct cost	
Total indirect cost	50% (2020) to 40% (2030) of direct cost	
Operating **Expenditures**—OPEX		
O&M cost	2.5% of direct cost	Adopted from Literature (Bertuccioli et al., 2014; Colella et al., 2014)
Stack replacement cost	20% of direct cost	Ranges between 5 and 40% (literature)
Water feedstock cost	A$5 kL^−1^ (desalinated water) Note: Comparison with other water sources is provided in Figure S3.	Adopted from literature (Shwisher et al., 2019)
Miscellaneous costs	2.5% of electrolyzer CAPEX per year	Assumed
**Electricity Consumption Cost:**		
Grid-operated scenario	Wholesale electricity pricing of the NEM was adopted. Price varies spatially between states and capacity factor of grid (Figure 3)	Refer to STAR Methods
Renewable PPA-operated scenario	PPA costs were evaluated at different WACCs and electricity market scenarios (Figure 2). For the base WACC case (6.25%), the following values are given: Solar PPA cost: A$47 MWh^−1^ (2020) – A$21 MWh^−1^ (2030) Wind PPA cost: A$51 MWh^−1^ (2020) – A$41 MWh^−1^ (2030)	
Stand-alone integrated operation scenario	The capital and operating costs of the solar and wind farm were added to the capital and operating costs of electrolyzer	

The parameters are elaborated in detail in the STAR Methods section.

#### Capital cost of Electrolyzer System—CAPEX

##### Direct costs

**Figure 2:**
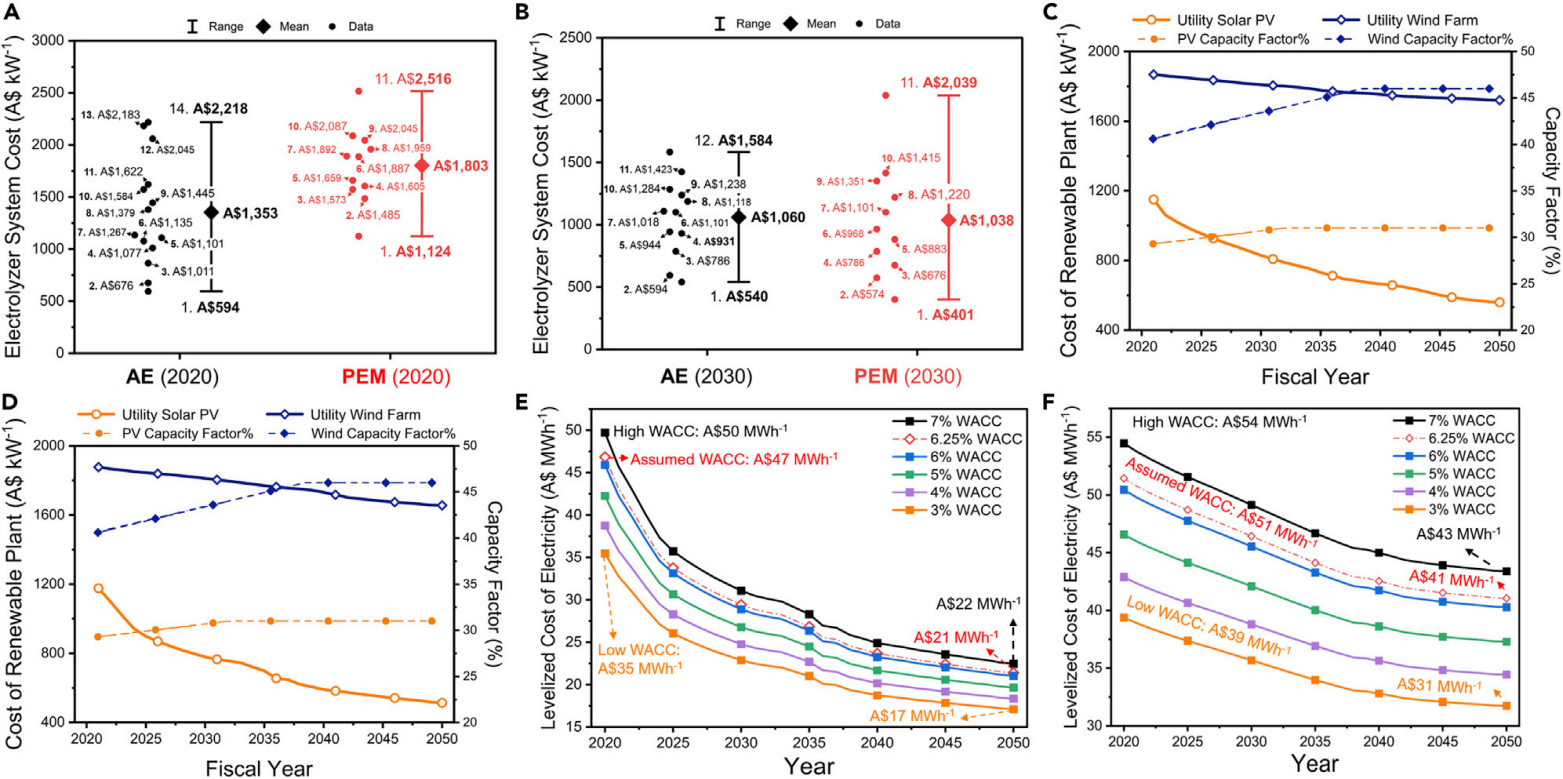
The projected purchase/build costs of the electrolyzer and renewable generator systems in Australia (A) Purchase cost of AE and PEM systems in 2020; (B) Purchase cost of AE and PEM systems in 2030; (C) Build cost/capacity factor of solar and wind farm for the central energy scenario; (D) Build cost/capacity factor of solar and wind farm for the high variable renewable energy scenario (HVRE); (E) Levelized costs of solar- and wind-based electricity under the central energy scenario for the assumed WACCs; (F) Levelized costs of solar- and wind-based electricity under the high variable renewable energy scenario for the assumed WACCs.The average purchase costs for electrolyzer systems for both AE and PEM systems were determined by collecting projected costs for 2020 (A) and 2030 (B) from literature, analysis reports, and correspondences with electrolyzer manufacturers (Table S3). In each year, these costs range between a high and low; the data points were arranged in ascending order (lower at the bottom and the highest on the top) to represent the cost range. The mean (simple arithmetic mean) of each of these ranges was used as an average representation of purchase costs, while the low and high costs were used as a measure of uncertainty. The difference of distance between the data points (black and red circles) is arbitrary and does not reflect anything; the data points were distributed to avoid overlap. Similarly, the cost of the renewable plant (solar and wind) was adopted from projections made by Graham et al., 2020 for electricity generators under the central energy scenario (low-cost reduction) and high variable renewable energy (high-cost reduction) scenario (Graham et al., 2020). We use the HVRE case for our analysis. These projected costs of solar and wind farms were then used to evaluate the represented levelized cost of electricity (LCOE). The LCOE, in turn, varies based on the assumed weighted average costs of capital; this is explained in detail in the STAR Methods section. The capital costs of solar PV and wind system and their LCOEs for each year are also enumerated in Tables S7 and S8, respectively.

Various literature studies have extensively explored and suggested trends for electrolyzer purchase costs for AE and PEM electrolyzer systems. However, each of these studies is based on an individual criterion, sources, and basis that results in a wide range of reported system costs. One of the most extensive analyses on electrolyzer costing was conducted by Bertuccioli et al., as part of the European Union’s Fuel Cells and Hydrogen Joint Undertaking, that developed long-term technical targets for electrolyzer systems for 2020 and beyond, based on data provided from manufacturers and stakeholders (Bertuccioli et al., 2014). The analysis reports an uninstalled average electrolyzer system with a cost of 630 €_2014_ kW^−1^ (range: 370–900 €_2014_ kW^−1^) and 1,000 €_2014_ kW^−1^ (range: 700–1,300 €_2014_ kW^−1^) for AE and PEM, respectively, in 2020, with the expectation that the costs can be reduced to 580 €_2014_ kW^−1^ for AE (370–800 €_2014_ kW^−1^) and to 760 €_2014_ kW^−1^ for PEM (250–1,270 €_2014_ kW^−1^) by 2030. A more recent assessment suggested uninstalled costs of AE systems to be between 570–1,500 €_2017_ kW^−1^ and 1,370 €_2017_ kW^−1^ for PEM system in 2020, reducing to a range of 787–906 €_2017_ kW^−1^ for AE and 397–955 €_2017_ kW^−1^ for PEM by 2030 (Sabaa et al., 2017). Glenk et al. suggest that the cost of the electrolyzer system is reducing at a rate of 2.96 ± 1.23% and 4.77 ± 1.88% per year for AE and PEM systems, respectively, strengthening the fact that PEM systems have the higher room for cost improvement (Glenk and Reichelstein, 2019). The major cost reduction is expected through research and development (R&D), leading to better system design that will benefit efficiency and scaling of production. Thus, in the short term, AE may be better suited for cost reduction arising from R&D and scaleup, but by 2030, PEM technology is expected to be a cost-effective option (Schmidt et al., 2017). We summarize the above-stated costs alongside other reported capital costs of electrolyzers, including those from the IEA (IEA, 2019), IRENA (IRENA, 2019), and Bloomberg Energy Finance (BNEF, 2019) and through direct correspondences with electrolyzer manufacturers to map out a range of probable electrolyzer costs. These costs are provided in a different currency and year basis (Table S3), and for consistency, we converted them to present-day Australian dollar basis (A$) by accounting for exchange rates and inflation (Table S4) to develop a range of cost for 2020 and 2030 (Table S5), as shown in Figure 2.

Based on the adopted cost projections, we calculate that the average cost of AE systems for 2020 to be A$1,353 kW^−1^ (range: A$594–2,516 kW^−1^) and expect it to decrease to A$1,060 kW^−1^ (range: A$540–1,548 kW^−1^) by 2030. At the same time, the PEM cost is expected to drop from an average cost of A$1,803 kW^−1^ (range: A$1,124–2,218 kW^−1^) in 2030 to $1,038 kW^−1^ (range: A$401–2,039 kW^−1^). Considering the large data range, we carry out the rest of our analysis with the average purchase cost for both AE and PEM systems. Additionally, the LC_H2_ is for the highest and lowest cost assumptions and is also provided to present a comprehensive and inclusive analysis.

##### Indirect cost of electrolyzer systems

It is, however, essential to acknowledge that the costs above are quoted as “uninstalled cost”, and these do not include the indirect cost of installing the electrolyzer. The uninstalled costs are highly subjective and tend to vary based on application and locations. However, to the best of our knowledge (Table S1), there are no defined indirect cost models for electrolyzers in Australia, presenting a significant research gap. Given that local electrolyzer supply chains in Australia are yet to be established, and there is an absence of local manufacturing capability, electrolyzer equipment would have to be imported and transported over large geographical distances at a cost (i.e., Siemens and ThyssenKrupp manufacturing units are situated in Germany) which would add to the overall capital costs. These indirect costs include the cost of importing the electrolyzers and the costs of preparing the installation site and licensing the facility. Initially (for projects starting in 2020), we assume that these indirect capital costs are equivalent to 50% of electrolyzer purchase costs, which are expected to decrease to 40% by 2030 (refer to STAR Methods)

This way, the overall capital cost of the project includes both the direct costs incurred by purchasing the electrolyzer and the indirect costs to develop the electrolyzer facility. Our analysis reveals that the direct costs would contribute to about 67% and 72% of the electrolyzer CAPEX in 2020 and 2030 respectively, while the indirect cost would contribute up to 33–28% of the CAPEX (2020–2030) (Figure S2).

#### Operating Cost of the Electrolyzer System—OPEX

For the operating expenses, we include feedstock costs for electricity and water, routine operation and maintenance (O&M) requirements, stack replacement costs, and other miscellaneous costs such as taxation, ongoing licensing, inflation, interest, etc., to develop a comprehensive cost model. Here, the electricity cost depends on the electrolyzer's operational configuration, which we have elaborated below. Besides, we considered desalinated water for hydrogen generation as most hydrogen projects in Australia are likely to be located close to the port to assist hydrogen export (Feitz et al., 2019) and shortage of fresh water in Australia due to prevailing drought conditions (Kiem, 2013). Yet, it is important to acknowledge that desalinated water is a costly resource, and less expensive water can be sourced from fresh resources and through water recycling (Shwisher et al., 2019). Nevertheless, our analysis (Figure S3) shows that the cost of water has a minimal impact on the overall LC_H2_; thus, water source would most likely be an operational and social decision rather than an economic concern.

The electrolyzer O&M costs are assumed to be equal to 2.5% per year of the electrolyzer purchase cost (Bertuccioli et al., 2014; Colella et al., 2014). The stack is considered to be replaced after 40,000–80,000 hr of cumulative operation at 20% of the uninstalled electrolyzer cost. The additional miscellaneous cost is assumed as 2.5% of the electrolyzer capital cost per year. Overall, the electricity consumption contributes ~30–60%, water consumption contributes ~1–6%, electrolyzer O&M contributes ~6–8%, miscellaneous costs contribute ~6–13%, and stack replacement contributes ~2% of the overall LC_H2_ (over 20 years).

#### Renewable electricity pricing and availability in NEM

There is a growing potential for low-cost electricity from solar PV and wind in the Australian electricity market. The wind and solar PV generation had already reached a ~21% share of the total electricity generated by the Australian grid (Commonwealth of Australia, 2020). This growth is being observed across Australia, with every state recording a significant increase in individual renewable energy capacity over the last decade (Commonwealth of Australia, 2020). Especially in South Australia, wind and solar PV are already contributing to over 50% of the total electricity generation. This significant growth in renewable energy then opens the avenue for leveraging Australia's vast renewable energy potential to generate green hydrogen using electrolysis.

To this end, we consider the expected costs of developing solar and wind farm projects as suggested by the AEMO (Graham et al., 2020) for developing solar and wind farms under a conservative central scenario (Figure 2C) and the high variable renewable energy scenario (HVRE, Figure 2D). The central scenario assumes a lower uptake of renewables resulting in a less acute reduction in solar PV and wind farm costs. In contrast, the HVRE scenario considers a rapid increase in renewable energy generation and subsequently sharper reduction in the renewable generator costs. Under the central scenario assumptions, the costs of utility-scale solar PV farms are projected as 1,150 A$ kW^−1^ (2021) that would decrease to 559 A$ kW^−1^ (2050) and similarly for onshore wind farms at 1,868 A$ kW^−1^ (2021), decreasing to 1,721 A$ kW^−1^ (2050). In contrast, for the HVRE scenario, the costs of solar farms would range between 1,150 A$ kW^−1^ (2021) and 531 A$ kW^−1^ (2050) and for wind farms between 1,868 A$ kW^−1^ (2021) and 1,656 A$ kW^−1^ (2050). Moreover, in each energy scenario, the increasing share of renewables will lead to higher availability of variable renewable electricity on the grid. This availability can be represented by the “capacity factor” of the solar and wind farms. For instance, the utility PV solar farm capacity factor is expected to increase from 29.5% (2021) to 31% (2050). Similarly, the capacity factor of wind farms is expected to increase to 46% by 2050, up from 41% in 2020. The AEMO expects to implement the HVRE scenario; there would be a need to develop flexible demand resources, a gap that electrolyzers can ideally fit into, given their intrinsic ability to operate at variable loads (Australian Electricity Market Operator, 2020).

Based on the capital and operating costs of the solar and wind farms provided by AEMO, we evaluated the levelized cost of electricity (LCOE) from solar and wind farms in each year between 2021 and 2050 as a potential scenario for electrolyzer deployment. Considering the current cost of capital (defined as WACC, 6.25%), the cost of electricity supply from the solar farms would be A$47 MWh^−1^ in 2021, declining to A$21 MWh^−1^ by 2050 (the red dotted line in Figure 2E). In contrast, wind electricity costs will be ~A$51 (2020), decreasing to A$41 MWh^−1^ by 2050 (the red dotted line in Figure 2F). However, we note that, if the WACC can be reduced to ≤5%, it may be possible to contract PPA costs of A$30 MWh^−1^ for solar and A$40 MWh^−1^ for wind as early as 2030, as opposed to those in 2040–2050 under current levels of WACC (6.25%). These low price points would be favorable for electrolyzer operation, despite the limited capacity factor. We explore these avenues further in scenario 1. For this analysis, we use a simplification by assuming that the WACC can be reduced to the suggested levels and do not analyze financing schemes required to achieve the suggested WACCs.

#### Electrolyzer participation in wholesale electricity markets

The electricity price will vary by time and location—a factor for siting such projects. A conventional approach to managing price risk is a long-term power purchase agreement that effectively locks in a fixed volume (MWh) and price ($/MWh) for the period of project financing. The volume will generally be matched to the electrolyzer capacity on a 24/7 basis, allowing operation at a very nominal output (MW). Such contracts will generally be priced according to the costs of providers providing assured 24/7 electricity for such large customers, as well as in consideration of other market factors, including future price expectations and risk premiums.

An alternative approach is for electrolyzer projects to take price risk as spot market purchasers. The key advantage here is that electricity market pricing can be quite volatile, with periods of very low (perhaps even negative) prices as well as potentially very high prices. As noted above, flexible electrolyzers could take advantage of such price volatility to operate only at times of lower prices. The tradeoff is, of course, lower overall capacity factors as the unit does not operate at high prices.

In our study, we present one possible approach for assessing the potential value of such flexible operation by electrolyzers using historical spot market pricing data from the NEM. One or more years of price data can be ordered in a “price duration curve” to provide an outlook of the different increases in costs of electricity for there is, of course, the challenge of what future electricity prices may actually be, and the produced hydrogen will involve emissions associated with the generation mix operating at the times the electrolyzer is running. One option for addressing both these issues is using PPAs with grid-connected wind and solar projects that can both lock in future electricity pricing for the electrolyzer (although not necessarily volume) and effectively ensure that the produced hydrogen is green. Moreover, operating electrolyzers with PPAs has an additional advantage as the electricity can still be bought from the grid, even if the contracted wind or solar projects are not generating electricity.

Given that electricity is always readily available on the grid due to the base load electricity being supplied by fossil-fuel-based power plants, the electrolyzer can now be operated at high capacity factors irrespective of the intermittent supply of the grid-connected solar and wind farms. However, as highlighted earlier, electricity pricing can be an issue, especially in markets such as the NEM, based on market-oriented electricity pricing arrangements that can see half-hourly wholesale prices ranging from -A$1,000 MWh^−1^ to over A$14,000 MWh^−1^ (AEMO data for 2018). In the absence of a centralized grid and pricing, each state in Australia has their own energy policy and hence fuel mixture for electricity generation. The wholesale electricity pricing has been rising year on year, as the fossil fuel generators (coal and gas) are aging, becoming less efficient, and costlier to operate primarily due to high fuel costs and uncertain availability (Australian Energy Market Regulator, 2018). Though recent times have seen an increasing share of renewable energy, especially with the growth of rooftop solar PV contribution to the grid, most of the capacity enhancement to meet the demand has been through induction of fossil fuel generators (Australian Energy Market Regulator, 2018; Australian Energy Regulator, 2020). Nevertheless, the availability of surplus renewables from distributed generation and the declining costs of utility-scale renewables have resulted in increasing time frames with negative electricity pricing.

### Benchmark case: Grid-operated electrolysis—high-capacity operation

**Figure 3:**
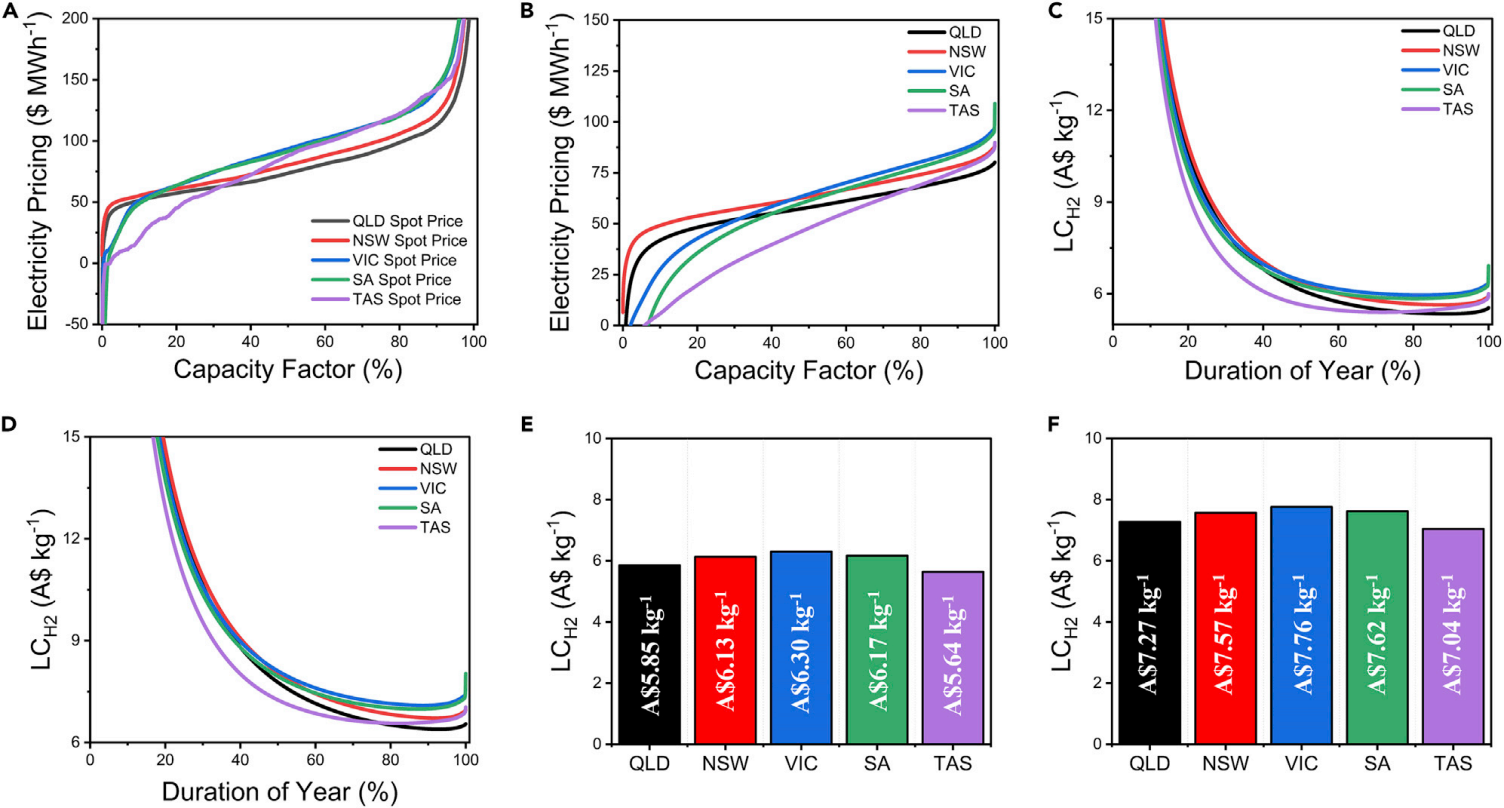
The outlook of levelized cost of generating hydrogen based on wholesale electricity pricing for grid-supplied electricity observed in the NEM for FY2018-19 (A) Statewise breakdown of the NEM spot price pricing; the prices experience volatility over the capacity factor of the grid; (B) The representation of the NEM spot pricing as an “average electricity price; (C) LCH_2_ distribution for the grid-operated AE system; (D) The LCH_2_ distribution for the grid-operated PEM system; (E) Statewise comparison of AE system LC_H2_ costs; (F) Statewise comparison of PEM system LC_H2_ costs. The spot price on the NEM fluctuates between different states as well as time; we collected the spot prices for the FY2018-19 and arranged them with the increasing capacity factor of the grid (elaborated in STAR Methods) to draw the shown duration curves. These duration curves can be represented as the actual spot price (A) or an average spot price (B), which is the cumulative spot price at any given point. These price duration curves highlight that operating the electrolyzer at the spot price exposes the electrolyzer to risk due to the fluctuation in pricing. However, operating with the average spot price minimizes the risk by leveling out the higher end costs by the lower end costs. Based on these costs, we calculated the levelized cost of generating hydrogen (LC_H2_) at each instant of the year for the corresponding electricity price to achieve the represented LC_H2_ duration curves. Given that the price duration varies between each state, we provided the individual costs for both AE (E) and PEM (F) in each state for a comparative analysis. A detailed comparison of electricity pricing between each state and the combined NEM is represented in Figure S4. Besides, the LC_H2_ distribution is represented at different truncated levels of capacity factor in Figure S5.

To analyze this scenario, we consider the NEM price duration curves for the FY2018-19 (Figures 3A and 3B) that provide the spot price and average wholesale electricity price over the duration of a year (July 2018–July 2019). During this time, the average spot electricity price within the NEM was reported to be ~A$100 MWh^−1^, but high price variability was observed with half-hourly wholesale price ranging from -A$1,000 MWh^−1^ to over A$14,000 MWh^−1^ (Australian Energy Regulator, 2020). These prices not only vary year by year and by region, and recent years have seen high average prices in Australia by historical standards for a range of reasons (AEMO, 2019). Notably, Victoria (VIC), which generally exhibits lower electricity pricing, in 2019 had the highest average pricing (A$126 MWh^−1^) due to several unscheduled power station outages. South Australia (SA) had the second highest pricing (A$125 MWh^−1^), despite the state reporting more negative price periods than the other states, reflecting its high wind penetration and only limited interconnection to the rest of the NEM. New South Wales also observed a slight increase in electricity pricing, reporting an LCOE of A$89 MWh^−1^. Tasmania also observed an unseasonal rise in electricity pricing (A$95 MWh^−1^) due to reduced rainfall putting stress on the lower cost electricity generation from hydropower. The lowest NEM pricing was reported in Queensland (A$75 MWh^−1^); the state shows a sustained decrease in electricity costs mostly driven by the enhancement of solar PV generation. A mix of electricity pricing exists in Australia that would translate into varying LC_H2_ pricing in the grid configuration.

To analyze this, we evaluated the LC_H2_ in each state based on the average wholesale electricity pricing in FY2018-19 (Figures 3C–3E). Surprisingly, it was Tasmania that offers the most attractive LC_H2_, A$5.64 kg^**−1**^ for AE and **A$**7.04 kg^**−1**^ for PEM, instead of Queensland; this is because Tasmania had longer durations with lower spot pricing than Queensland that led to overall lower average wholesale pricing in Tasmania as compared to Queensland. This anomaly can be explained by observing the distribution of the LC_H2_ across the whole year (Figures 3C and 3D). Based on the capacity factor and how the average electricity price varies over the year, the levelized cost would be a distribution. For instance, for a grid capacity factor of 20%, the levelized cost is very high (>A$15 kg H_2_^−1^ for both AE and PEM), arising from lower capital efficiencies of the electrolyzer, despite very low electricity pricing. When the capacity factor >40%, we note that the LC_H2_ tend to flatten off owing to the tradeoff between capital efficiency and increased cost of electricity consumption before rising again as the capacity factor approaches 100%. This trend is interesting and counter intuitive as initially it may seem that operating the electrolyzer at times when electricity pricing is low would lead to lower LC_H2_, whereas, in fact, it is more beneficial to run the electrolyzer for longer durations despite increasing operating costs as it would lead to overall capital and operating expenses being levelized over larger amounts of hydrogen generated.

The grid-operated scenario for electrolyzers was also evaluated as part of the Australian National Hydrogen Roadmap. Their analysis suggested that the LC_H2_ would range between ~ A$4.78 kg^−1^ and $5.85 kg^−1^ for AE systems and between A$6.08 kg^−1^ and $7.43 kg^−1^ for the PEM systems (Bruce et al., 2018). While these costs are within the range we proposed, it must be noted that the Roadmap results were suggested for A$60 MWh^−1^ at an 85% capacity factor.

### Solar/wind PPA operated electrolysis

#### Influence of intermittent operation

The other scenario we considered is operating the electrolyzer with exclusively sourced renewable solar and wind electricity by negotiating PPAs with the grid-connected solar PV and wind farms. It is assumed that, by locking in the PPAs, the electrolyzer will be able to source electricity at the LCOE generated by the connected solar or wind farm. Given that the LCOE of new upcoming solar and wind farms is expected to decrease (Figure 2), the costs of PPAs will also fall as future solar PV and wind farms come online. Simultaneously, the capital cost of electrolyzers is also decreasing every year; combined with the lower PPA pricing, these cost reductions would add up to significantly reduce the LC_H2_. To analyze this, we calculated the levelized cost of generating hydrogen for investment windows, as suggested earlier. Window 1 (2020–2040) considers that the electrolyzer is assumed to be installed and financed under the 2020 costs and performance parameters. In contrast, window 2 (2030–2050) considers a future scenario where the electrolyzers are installed and financed under the 2030 assumptions. Overall, window 1 has significant importance as it provides an analysis on how investment in the near term will dictate LC_H2_, while the analysis for window 2 provides insights on how LC_H2_ can potentially decrease in the long term.

For window 1 (2020–2040), we calculate that the AE and PEM systems have an installed capital cost of A$20.3 million and A$27.0 million, respectively, leading to a present value annuitized CAPEX costs of A$1.8 million per year (AE) and A$2.2 million per year (PEM). Similarly, the electrolyzer facility operated in window 2 (2030–2050) will have an installed capital cost of A$14.3 million for AE and A$14.0 million for PEM, leading to annuitized CAPEX costs of A$1.3 million per year (AE) and A$1.2 million per year (PEM). As expected, the CAPEX for developing the PEM system becomes more favorable than the AE system after 2030. Moreover, the efficiency (SEC) of both electrolyzer systems is expected to be at par by 2030 (Table S6). These factors translate into the levelized cost of hydrogen as well, as the PEM system operated in window 2 becomes more competitive with the AE system for both solar and wind PPA configuration (Figure 3). Our analysis reveals an average LC_H2_ with a solar-powered AE system of A$7.0 kg^−1^ in window 1 and A$5.3 kg^−1^ in window 2, respectively. In contrast, the average LC_H2_ for the PEM system in window 1 is A$8.9 kg^−1^ and is expected to decline to A$5.0 kg^−1^ in window 2 owing to a higher rate of decrease for PEM purchase costs (A$ kW^−1^) and higher projected efficiency improvement (kg_H2_ kWh^−1^). Similarly, the average cost of the wind-powered AE system will be A$6.2 kg^−1^ in window 1 (2020–2040), decreasing to A$5.0 kg^−1^ in window 2 (2030–2050). In comparison, the wind-powered PEM system will present a levelized cost of A$7.3 kg^−1^ in window 1, which decreases to A$4.6 kg^−1^ in window 2. These average LC_H2_ values are representative of the hydrogen supply costs over the 20-year project life.

To analyze the impact of the individual cost components on the LC_H2_, we break down the total LC_H2_ over the 20-year project life into individual LC_H2_ for each (Figure 3). Moreover, we break down the LC_H2_ in each year to represent the share of individual cash flows (capital cost recovery, operating costs, etc.) in the total LC_H2_. Our analysis shows that the major cost drivers are the electrolyzer capital recovery, i.e., the annuitized cost to recover the invested capital over 20 years (WACC: 6.25%) and the operating costs mainly due to electricity consumption (Figures S6 and S7). Another important factor to note is the electrolyzer degradation, which would lead to a lower amount of hydrogen produced than the preceding year. Thus, the production costs (capital recovery and operating costs) in every subsequent year have to be levelized over a smaller amount of hydrogen, leading to increased LC_H2_ (A$ kg^−1^). A large spike in LC_H2_ is observed when the stack is replaced, leading to additional operating costs. In practice, the cost of stack replacement is usually financed as additional capital costs or yearly operating costs (total cost annualized over project life). However, we represent it as a single cash flow for a more direct comparison with other cash flows (capital recovery, electricity costs, etc.) and highlight the importance of stack replacement costs. Another aspect is the practicality of stack replacement, given that the stack life is expected to range between 40,000 and 80,000 hr of operation (Bertuccioli et al., 2014; Schmidt et al., 2017); the stack would have to be replaced after 15 years for the solar PPA and 11 years for the wind PPA for 30% and 41% capacity factor, respectively, and stack life of 40,000 hr. This mismatch would mean that the new stacks operate for just 5 and 9 years, respectively, before the project is decommissioned, which is impractical. However, the costs of these underutilized stacks could be recovered by reselling the stack to the original equipment manufacturer (OEM) or other consumers as a salvage cost. We do not investigate this in our analysis, but our results show that replacing the stack would be beneficial given the future stack systems will have enhanced efficiency that leads to lower LC_H2_, that is, reflected in the reduced LC_H2_ after stack replacement, as the new more efficient stack generates more hydrogen. For example, in the solar PPA case, the reduced LC_H2_ reduces to A$6.0 kg^−1^ (AE)/A$7.6 kg^−1^ (PEM) as compared to the original stack, which gave an LC_H2_ of A$7.1 kg^−1^ (AE)/$8.8 kg^−1^ (PEM). The LC_H2_ curve, however, then retakes an upward trend as the electrolyzer begins to degrade again and lose its efficiency. If the increased stack life of 60,000 or 80,000 hr is achieved, the stack replacement would be due in ~15–16 years and 22–33 years, well beyond the project life. Thus, there would be no need for stack replacement, and the LC_H2_ will decrease. However, this results in a tradeoff with the degradation rate, as without stack replacement, the stack would degrade by up to 20% (for 1% degradation in output per year), increasing the total project life of LC_H2_ by ~2%.

**Figure 4:**
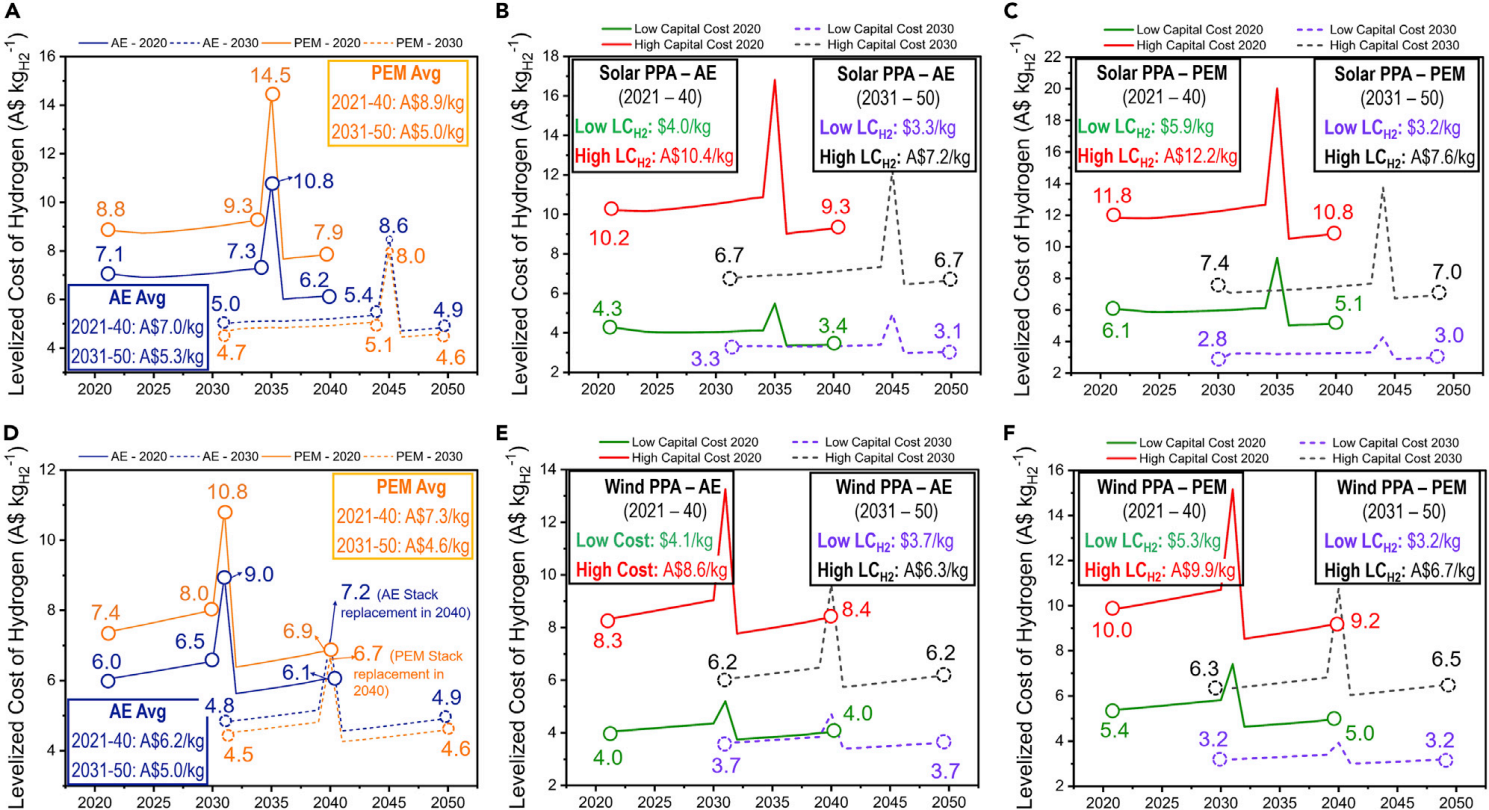
The annualized LC_H2_ profile for solar- and wind-powered AE and PEM electrolyzer systems as a function of the electrolyzer capital costs and PPA pricing for projects in window 1 (2021–2040) and window 2 (2031–2050) (A) LC_H2_ of the solar PPA-operated AE and PEM systems at the average electrolyzer CAPEX; (B) LC_H2_ of the solar PPA-operated AE system at the low and high electrolyzer CAPEX assumptions; (C) The LC_H2_ of the solar PPA-operated PEM system at the low and high electrolyzer CAPEX assumptions; (D) LC_H2_ of the wind PPA-operated AE and PEM systems at the average electrolyzer CAPEX assumptions; (E) LC_H2_ of the wind PPA-operated AE system at the low and high electrolyzer CAPEX assumptions; (F) The LC_H2_ of the wind PPA-operated PEM system at the low and high electrolyzer CAPEX assumptions. We show the levelized costs for the average CAPEX (A, D) as an average representation of LC_H2_ in each window for both the solar- and wind-powered cases. The LC_H2_ trends for the higher and lower electrolyzer capital cost assumptions are also represented (B, C, E, F). In these figures, the solid lines represent the LC_H2_ for window 1, and the dashed lines represent the LC_H2_ for window 2. The navy blue line represents the AE system, and the orange line represents the PEM system, while green and red lines represent the low and high capital cost case in 2020, respectively, and the dashed black and purple lines represent the low and high capital cost cases in 2030, respectively. The LC_H2_ quoted in the boxes represents the LC_H2_ over the total 20-year project life; the values are summarized in Table 2. The individual components of the cash flows are shown in Figures S6 and S7. Special focus was placed on the effect of efficiency and degradation as highlighted in the text and represented in Figures S8–S10.

Another important aspect is the CAPEX range (depending on the highest and lowest electrolyzer costs – A$ kW^−1^); for the solar-powered AE system in 2020, the average LC_H2_ could lie between A$4.0 kg^−1^ (A$594 kW^−1^) and A$10.4 kg^−1^ (A$2,218 kW^−1^) (Figure 4B), and for PEM, it could be between A$5.9 kg^−1^ (A$1,124 kW^−1^) and A$12.2 kg^−1^ (A$2,516 kW^−1^) (Figure 4C). By 2030, this range can shrink from A$3.3 kg^−1^ (A$540 kW^−1^) to A$7.2 kg^−1^ (A$1,584 kW^−1^) for AE and from A$3.2 kg^−1^ (A$401 kW^−1^) to A$7.6 kg^−1^ (A$2,039 kW^−1^) for PEM. Overall, the total project life of LC_H2_ close to ~ A$3 kg^−1^ can be achieved with solar PPA costs in window 2 (operation beyond 2030) if the low costs of electrolyzers (A$540 kW^−1^ for AE and A$401 kW^−1^ for PEM) can be achieved. For the same considered CAPEX ranges, the possible LC_H2_ of wind PPA-powered systems can be between A$4.1 kg^−1^ (A$594 kW^−1^) and A$8.6 kg^−1^ (A$2,218 kW^−1^) for AE system (Figure 4E) and between A$5.3 kg^−1^ (A$1,124 kW^−1^) and A$9.9 kg^−1^ (A$2,516 kW^−1^) for PEM system (Figure 4F). By 2030, this LC_H2_ range can vary between A$3.7 kg^−1^ (A$540 kW^−1^) and A$6.3 kg^−1^ (A$1,584 kW^−1^) for AE and between A$3.2 kg^−1^ (A$401 kW^−1^) and A$6.7 kg^−1^ (A$2,039 kW^−1^) for PEM. Note that the total project life of LC_H2_ close to ~ A$3 kg^−1^ can also be achieved with wind PPA during operation in window 2 (operation beyond 2030) for the low costs of electrolyzers (A$540 kW^−1^ for AE and A$401 kW^−1^ for PEM). Nevertheless, the wind-powered system is more viable than the solar-powered system in the long run, due to the higher capacity factor (41% compared to 31% of solar), leading to higher amounts of hydrogen generated at similar costs. These results are summarized in Table 2.

**Table 2:** The LC_H2_ outlook of PPA-powered electrolyzer scenario.

Scenario	Window #1: 2021–2040		Window #2: 2031–2050	
	Electrolyzer CAPEX (A$ Million)	LC_H2_ (A$ kg^−1^)	Electrolyzer CAPEX (A$ Million)	LC_H2_ (A$ kg^−1^)
Solar PPA scenario—AE system	Low_2020_: 8.9 (A$594 kW^−1^)	4.0	Low_2030_: 7.6 (A$540 kW^−1^)	3.3
	Avg_2020_: 20.3 (A$1,353 kW^−1^)	7.0	Avg_2030_: 14.8 (A$1,060 kW^−1^)	5.3
	High_2020_: 33.3 (A$2,218 kW^−1^)	10.4	High_2030_: 22.2 (A$1,584 kW^−1^)	7.2
Wind PPA scenario—AE system	Low_2020_: 8.9 (A$594 kW^−1^)	4.1	Low_2030_: 7.6 (A$540 kW^−1^)	3.6
	Avg_2020_: 20.3 (A$1,353 kW^−1^)	6.2	Avg_2030_: 14.8 (A$1,060 kW^−1^)	5.0
	High_2020_: 33.3 (A$2,218 kW^−1^)	8.6	High_2030_: 22.2 (A$1,584 kW^−1^)	7.3
Solar PPA scenario—PEM system	Low_2020_: 16.9 (A$1,124 kW^−1^)	5.9	Low_2030_: 5.6 (A$401 kW^−1^)	3.2
	Avg_2020_: 27.0 (A$1,803 kW^−1^)	8.9	Avg_2030_: 14.5 (A$1,038 kW^−1^)	5.0
	High_2020_: 37,7 (A$2,516 kW^−1^)	12.2	High_2030_: 28.5 (A$2,039 kW^−1^)	7.6
Wind PPA scenario—PEM system	Low_2020_: 16.9 (A$1,124 kW^−1^)	5.3	Low_2030_: 5.6 (A$401 kW^−1^)	3.2
	Avg_2020_: 27.0 (A$1,803 kW^−1^)	7.3	Avg_2030_: 14.5 (A$1,038 kW^−1^)	4.6
	High_2020_: 37.7 (A$2,516 kW^−1^)	9.9	High_2030_: 28.5 (A$2,039 kW^−1^)	6.7

The table summarizes the capital cost of the electrolyzer system in each window analyzed. Low represents the lowest capital costs, avg represents the average capital costs, and high represents the highest capital costs of electrolyzer in each window. The capital costs include both the purchase cost of electrolyzer (direct) and indirect costs, as shown in Table 1.

Note that as suggested earlier the cost profiles in Figure 4 represent the annualized levelized cost of generating hydrogen over the 20-year project life considered. In most regards, of course, the levelized costs are best presented on a project lifetime basis. However, presenting annualized levelized costs for projects as done here does provide some valuable insights on expected project cash flows and major expenditure points over the project life. It also highlights the impact of electrolyzer stack degradation on project returns and the potential risks of mismatching stack replacement lifetimes with the balance of plant lifetimes when electrolyzers are operated at lower capacity factors due to their integration with renewable generation options. Figure 4 also particularly highlights the impact of projected technology improvements and cost reductions for projects built now (2021) and those built in a decade's time. For example, for the solar PPA-powered AE system installed in the 2020 window, we estimate an overall LC_H2_ (20-year H_2_ supply cost) of A$7.0 kg^−1^ that decreases to A$5.3 kg^−1^ for investment in 2030; this cost reduction is mainly driven by the anticipated decline in capital costs between the two investment windows.

### Key to H_2_ below A$2 kg^−1^

**Figure 5:**
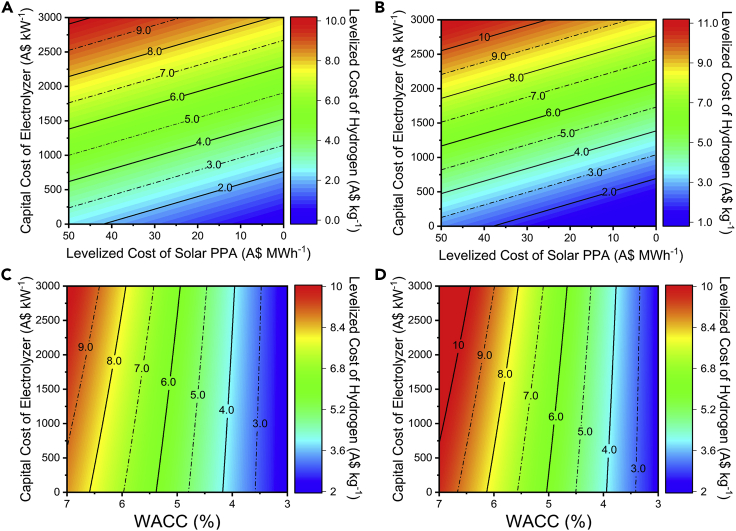
Contour map of LC_H2_ using a range of market conditions for solar PPA-powered electrolyzers (A) The LC_H2_ of the solar PPA—AE-powered system at different electrolyzer capital costs; (B) LC_H2_ of the different solar PPA—PEM-powered system at different electrolyzer capital costs; (C) LC_H2_ of the solar PPA-powered AE electrolyzers at different WACCs; (D) LC_H2_ of the solar PPA-powered PEM electrolyzers at different WACCs. The LC_H2_ of the AE and PEM system (A, B) is represented as a function of different electrolyzer capital costs—A$ kW^−1^ (direct + indirect cost)—and solar PPA pricing—A$ MWh^−1^—at a capacity factor of 31% (refer to STAR Methods). Similarly, the LC_H2_ of AE and PEM system is also represented (C, D) as a function of electrolyzer capital costs and different weighted average costs of capital at A$47 MWh^−1^ of solar PPA at a capacity factor of 31%. The figures show that to reach LC_H2_ below A$2 kg^−1^, the AE system capital cost would have to be reduced below A$500 kW^−1^ and operated ≤ A$20 MWh^−1^ (capacity factor of 31%) or the WACC of the electrolyzer would have to be below 4% (for solar, PPA cost of A$47 MWh^−1^ at a capacity factor of 31%).

We also carried out optimization investigations to identify parameters that can lead to low-cost hydrogen production. With the government target in mind, we determine the required electrolyzer capital costs (purchase plus indirect costs), solar PPA pricing (at a fixed capacity factor of 30%), and cost of capital (WACC) that could lead to the LC_H2_ ≤ A$2 kg^−1^ (Figure 5). To achieve this, the solar PPA-powered AE and PEM systems would have to be operated with PPA pricing available at ≤ 20 A$MWh^−1^, the electrolyzer CAPEX below A$500 kW^−1^, and the electrolyzer costs financed at a WACC <3.5%. Note that the electrolyzer capital costs are projected to reach these levels within 2030. However, the required electricity costs can only be achieved after 2050 if solar projects are financed at current WACC levels (6.25%). Nevertheless, it is plausible to achieve these costs by 2030 if WACC can be lowered to <4% (Figure 2E).

**Figure 6:**
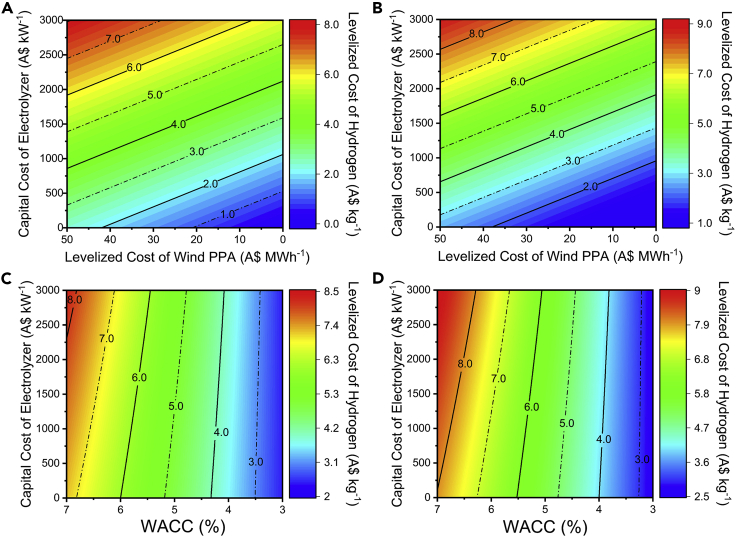
Contour map of LC_H2_ using a range of market conditions for wind PPA-powered electrolyzers (A) The LC_H2_ of the wind PPA—AE-powered system at different electrolyzer capital costs; (B) LC_H2_ of the wind PPA—PEM-powered system at different electrolyzer capital costs; (C) LC_H2_ of the solar PPA-powered AE electrolyzer at different WACCs; (D) LC_H2_ of the solar PPA-powered PEM electrolyzer at different WACCs. The LC_H2_ of the AE and PEM system (A, B) is represented as a function of different electrolyzer capital costs—A$ kW^−1^ (direct + indirect cost)—and solar PPA pricing—A$ MWh^−1^—at a capacity factor of 31% (refer to STAR Methods). Similarly, the LC_H2_ of AE and PEM system is also represented (C, D) as a function of electrolyzer capital costs and different weighted average costs of capital at A$51 MWh^−1^ of solar PPA at a capacity factor of 31%. The figures show that to reach LC_H2_ below A$2 kg^−1^, the AE system capital cost would have to be reduced below A$500 kW^−1^ and operated ≤ A$30 MWh^−1^ (capacity factor of 41%) or the WACC of the electrolyzer would have to be below 3% (for solar, PPA cost of A$51 MWh^−1^ at a capacity factor of 41%).

The wind PPA configuration would also require a similar optimal mix of PPA pricing, electrolyzer cost, and financing schemes to meet the Australian government target. To achieve LC_H2_ costs close to A$2 kg^−1^ would at least require electrolyzer capital costs (purchase plus indirect cost) below A$500 kW^−1^ with the wind PPA pricing of A$30MWh^−1^ (capacity factor of 41%) or WACC ≤3% (Figure 6). As suggested earlier, the electrolyzer costs can reach these levels by 2030, and significant focus would still have to be placed on reducing the PPA pricing or creating a favorable financing scheme. The required wind PPA pricing is projected to be achieved by 2050 under the current WACC breakdown (6.25%) and projected wind farm capital costs by 2030. Given these projections, near term wind farm projects would have to be financed below 4% WACC to achieve the A$2 kg^−1^ LC_H2_ target. For the electrolyzer systems, the capital costs are already on a downward trend. However, further reduction in LC_H2_ can also be achieved by increasing electrolyzer efficiency (Figure S8) or lowering electrolyzer degradation rates (Figures S9 and S10). In comparison, the cost of renewable projects can benefit from less stringent financing schemes that can be influenced by a combination of policies such as additional government subsidies or lower interest rates on debts. We further note that by increasing the contracted PPA's capacity factor, the LC_H2_ can be reduced (Figure S11).

Longden et al. have also provided an outlook on renewable electrolysis using grid solar and wind electricity in Australia (Longden et al., 2020). Their analysis estimated that the LC_H2_ can be reduced below A$3 kg^−1^ for electrolyzer CAPEX < A$1,000 kW^−1^ if the electricity price is A$32 MWh^−1^ (at 30% capacity factor) or A$46 MWh^−1^ (at 45% capacity factor). Our analysis, however, reveals that for A$3 kg^-^1, the capital cost would have to be decreased to A$750 kW^−1^ (including indirect costs) for an electricity price of $15 MWh^−1^ (at 30% capacity factor) or $25 MWh^−1^ (at 45% capacity factor). We note that these differences arise from the consideration of additional parameters within our framework, and this is discussed in depth in Table S1.

Our findings suggest that if the PPA can be priced at A$<40 MWh^−1^ for 70% of the year (electrolyzer capital cost of A$1,000 kW^−1^), the LC_H2_ can decline to reach A$2 kg^−1^. A major driver of capital cost reduction will likely be achieved through economies of scale. Though for this analysis, we do not analyze the effect of scale beyond that of 10MW plant sizing; Other works, including those by Bohm et al. (Böhm et al., 2020) and Proost (Proost, 2020), have highlighted the potential cost reductions available through a greater scale. In particular, Proost's analysis suggests that alkaline electrolyzer costs of €500 kW^−1^ (A$800 kW^−1^ exclusive of indirect costs) are possible with scaling electrolyzer capacity (50–100 MW), which would make these systems compatible with fossil fuel hydrogen supply if electricity can be sourced at €30 MWh^−1^ (A$46 MWh^−1^) at a capacity factor. However, our results highlight that if the indirect costs, degradation, etc. are considered, the electricity would have to be reduced to < A$40 MWh^−1^ and provided for 70% of the year to achieve parity with fossil-fuel-based hydrogen generation. These findings further signify the importance of considering a holistic model to establish cost benchmarks.

### Stand-alone renewable electrolysis

#### Influence of oversizing and downsizing

As noted earlier, another possible operational configuration of the H_2_ electrolyzer plant is to integrate onsite renewable electricity generation from solar PV or wind. Besides, the usage of off-grid renewable would enable the plant to operate in regions where the potential to generate solar and wind electricity is high. In Australia, some of the most prospective renewable generation areas are quite remote (Feitz et al., 2019). Yet, these regions offer a potential business case for using electrolyzers to generate hydrogen as a storage medium for excess renewables, and the hydrogen can then be used to generate electricity when there is no renewable supply or used domestically by blending with natural gas to create a versatile energy network.

**Figure 7:**
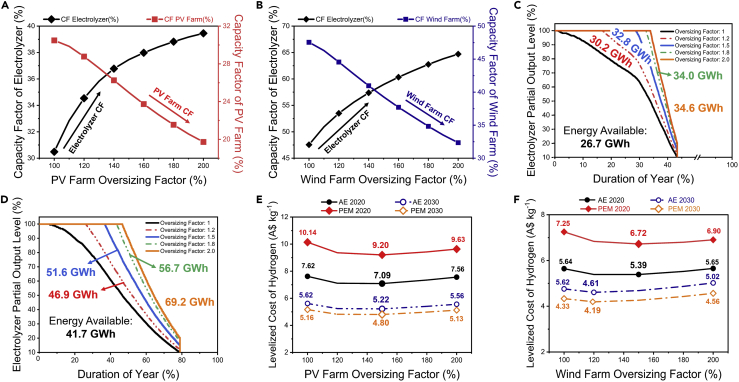
Impact of oversizing the dedicated solar and wind farm on the economics of the electrolyzer system (A) The tradeoff between the electrolyzer capacity factor and the solar PV farm capacity factor at different oversizing factors; (B) Tradeoff between the electrolyzer capacity factor and the wind farm capacity factor at different oversizing factors; (C) The increase in energy available to the electrolyzer at the different oversized capacity of solar PV farm; (D) The increase in energy available to the electrolyzer at the different oversized capacity of the wind farm; (E) The LC_H2_ of AE and PEM system under different oversized solar PV farm electricity supply; (F) The LC_H2_ of AE and PEM system under different oversized wind farm electricity supply. As observed, the capacity factor of the electrolyzer system increases by oversizing the solar PV or wind farm (A, B). Oversizing the solar PV and wind farm also increases the energy available to operate the electrolyzer (C, D). The oversizing also causes the LC_H2_ to decrease, with the lowest LC_H2_ (A$7.09 kg^−1^ for AE and A$9.20 kg^−1^ for PEM) achieved at the oversizing factor of 150% for solar PV (E) and similarly for 150% oversized wind (A$7.09 kg^−1^ for AE and A$9.20 kg^−1^ for PEM [F]) in 2020. For 2030, the LC_H2_ is the lowest for 150% oversized solar but with 120% oversized wind (reason detailed in text). Here, the oversized factor is the percentage ratio of solar PV/wind farm capacity (MW) to electrolyzer capacity (MW). The duration curve of the solar and wind farm represented here is based on the Moore Solar Farm and Kiata Wind Farm (Figure S12). These results are compared with oversizing the solar and wind farms based on duration curves of other solar and wind farms across Australia in Figure S13. In the LC_H2_ figures, the solid lines represent the LC_H2_ for AE- (black) and PEM (red)-integrated system assumed to be constructed in 2020, whereas the dotted lines represent the LC_H2_ for AE (blue) and PEM (orange) systems considered to be built in 2030.

We evaluated the LC_H2_ of this configuration (Figure 7) for different capacity stand-alone solar/wind farms (10–20 MW) integrated with a 10 MW AE and PEM electrolyzer system. As mentioned earlier, a similar capacity PV/wind farm (10 MW) would only operate the electrolyzer for a capacity factor of 30–47%, respectively. However, oversizing the renewable plant (Figures 7A and 7B) would then allow the electrolyzer to operate at a higher capacity factor; for instance, a solar or wind farm with a capacity 1.4 times higher than the electrolyzer (14 MW) can operate the same electrolyzer system for 37% and 57% capacity factor, respectively. Similarly, a 20 MW electricity generator would operate the electrolyzer (10 MW capacity) at up to 43% and 67% capacity factor. This approach would have a significant advantage as the electrolyzer driven with a larger renewable energy generator would have higher energy available to generate H_2_ than a similar capacity generator. For example, the 10 MW solar and wind plant can only provide 26.7 GWh and 41.7 GWh of energy, respectively, to the electrolyzer. In comparison, at 20 MW, solar and wind energy generators can provide up to 34.6 GWh and 69.2 GWh, respectively, a 30% (solar) and 60% (wind) increase in available energy (Figures 7C and 7D). However, this increase in the available power comes at the cost of additional CAPEX and OPEX of the renewable generator creating an interesting tradeoff that subsequently affects the LC_H2_. As seen in Figures 7E and 7F, the correlation of the AE/PEM electrolyzer LC_H2_ as a function of increasing the capacity of the renewable energy generator follows a U-shaped curve. To be specific, the LC_H2_ starts at a high value (when electrolyzer and renewable generator is of the same capacity), declining to the lowest value when the renewable generator is oversized before increasing again. The above behavior occurs because, initially, there is a sharper increase in the electrolyzer's capacity factor per marginal MW increase in the renewable generator; leading to an increase in hydrogen generation occurs, offsetting the subsequent increase in capital and operating costs arising from oversizing the renewable generator and eventually leading to an overall lower LC_H2_. However, after a specific capacity (1.5 times electrolyzer capacity), further increase in the renewable generator power leads to a significant curtailment, and the improvement in the electrolyzer capacity factor starts to level off.

Similar to the PPA scenario, here, we also evaluated the LC_H2_ for systems installed in 2020 (operating from 2021 to 2040) and the systems installed in 2030 (operating from 2031 to 2050). The analysis assumes the electrolyzer average capital cost for both 2020 (A$2,029 kW^−1^ for AE and A$2,704 kW^−1^ for PEM) and 2030 (A$1,484 kW^−1^ for AE and A$1,452 kW^−1^ for PEM), including indirect costs. For a 10 MW solar PV farm-powered electrolyzer installed in 2020, we observe an LC_H2_ of A$7.62 kg^−1^ for the AE system and A$10.14 kg^−1^ for the PEM system (Figure 7E). The lowest costs are achieved at a 15 MW solar PV farm capacity, with the LC_H2_ for the AE and PEM systems declining to A$7.09 kg^−1^ and A$9.2 kg^−1^, respectively. Similarly, for a 10 MW wind farm-powered electrolyzer, the LC_H2_ is calculated to be A$5.39 kg^−1^ for the AE system and A$6.72 kg^−1^ for the PEM system (Figure 7F), with the lowest costs achieved at around 15 MW capacity where the LC_H2_ declines to A$5.39 kg^−1^ for AE and A$6 kg^−1^ for PEM. These LC_H2_ are represented for the integrated solar/wind farm-electrolyzer facility developed in 2020, and with the improvement in the electrolyzer and renewable generator costs by 2030 (Table S7), the subsequent LC_H2_ can decrease further. For 2030, the solar-powered configurations would still be most optimum at 15 MW capacity with an LC_H2_ of A$5.2 kg^−1^ for AE and A$4.8 kg^−1^ for PEM, while the wind-powered configuration would be optimum at a lower 12 MW capacity (120% oversizing ratio) with an LC_H2_ of A$4.6 kg^−1^ for AE and A$4.2 kg^−1^ for PEM. This difference in optimum oversizing ratios of wind systems is because, by 2030, the electrolyzer capital costs (~A$1,400 kW^−1^) are significantly lower than the cost of the wind system (A$1,800 kW^−1^). Thus, the cost of the wind system dominates the LC_H2_, and oversizing the wind system causes the LC_H2_ to increase more significantly compared to that in 2020, where the cost of both systems is similar.

Moreover, given that our model is scale independent beyond our assumptions based around 10 MW unit sizes, the electrolyzer and solar/wind farm (MW) capacity does not need to be formally specified. As such, either oversizing the renewables to the electrolyzer capacity or undersizing the electrolyzer to the renewable generator capacity is equivalent provided they are oversized or undersized at the same ratio. Note, however, that large-scale electrolyzer systems are expected to be less expensive to build (A$ kW^−1^) than their lower capacity counterparts (Böhm et al., 2020; Proost, 2020). Similarly, the cost of renewables solar and wind also vary based on their scale; thus, combining these systems with the electrolyzer at different capacity scales would create an interesting tradeoff. Moreover, given that the costs of building solar and wind farms (A$ kW^−1^) are different from those of the electrolyzer (A$ kW^−1^), it would be overall economically viable to keep the scale (subsequently cost) of the more expensive system constant and optimize the cost of the low-cost system. As highlighted earlier, we did not consider scaling within our framework, given that the electrolyzer costs are not expected to increase significantly beyond the 10 MW scale (Bruce et al., 2018) and present uncertainties on how the costs will differ with scaling (Böhm et al., 2020). However, this is a limitation of our model and should be considered in future work.

Several other works have explored stand-alone electrolysis systems. Hinkley et al. also considered a stand-alone PV-powered PEM electrolyzer in Australia, suggesting a high LC_H2_ of ~A$9 kg^−1^ in 2030 (electrolyzer capital cost of A$1,200 kW^−1^, PV capex of A$1,412 kW^−1^) (Hinkley et al., 2016). However, this analysis considered small-scale system (950 kW) and low-capacity factors (20%). With an oversized PV system, we are able to increase the capacity factor of our electrolyzer to 39% and match the LC_H2_ despite considering a high capital cost. More recently, Yates et al. analyzed a stand-alone solar PV-powered electrolyzer system, and they suggested a levelized cost of A$4.45–5.18 kg^−1^ for Australia (Yates et al., 2020). They considered electrolyzer costs between A$900 and 1,120 kW^−1^ and solar PV costs of A$1,150 kW^−1^. However, they specifically considered sites in Townsville, Queensland, Broome, and Western Australia, with high solar potential to achieve solar capacity factors of 34%–38%. Our analysis suggests that the current cost of stand-alone PV systems will range between A$8 and 10 kg^−1^ at an average solar capacity of 30% and between A$7 and 9 kg^−1^ if the solar capacity factor can be optimized to 35% (electrolyzer capital cost: A$1,353 (AE) – 1,803 kW^−1^ (PEM) and solar PV cost of A$1,285 kW^−1^). Even if we consider the same cost assumptions as in their analysis (Yates et al.), we achieve the best-case cost of A$5.31 kg^−1^; the major difference is the inclusion of indirect costs. If we exclude our assumption of indirect costs, the LC_H2_ fall in the range specified by Yates et al., further signifying the importance of including indirect costs. Moreover, they also explore oversizing of the solar farm, finding oversizing ratio of 1.5 to be the most optimum; however, we suggest that 1.4 is the most optimum ratio. We also extended the oversizing to the stand-alone wind-operated scenario and explored the potential for undersizing the electrolyzer.

## Discussion

### Addressing curtailment

The downside of the oversized dedicated renewable supply to the electrolyzer is that in the absence of an additional load for the surplus energy produced, the oversized renewable generator must be curtailed. This leads to spillage of usable renewable energy, subsequently reducing the capital efficiency of the solar and wind farms. A potential strategy to address this could be that instead of curtailing, the surplus can be retailed, and the revenue generated would then offset the additional cost of developing and operating the solar/wind farm that is passed on directly to the levelized cost of generating hydrogen in the stand-alone scenario. Moreover, instead of curtailing, the excess energy can also be used to operate systems downstream like a compressor for injecting hydrogen into the natural gas grid, operating a hydrogen refueling station, or for subsequent conversion of hydrogen into ammonia, methanol, etc. These opportunities should be considered to design future solar and wind farms that can be specifically oversized to operate electrolyzer at high capacities as a primary load while the excess electricity is sold to the grid. Alternatively, the surplus electricity could be stored onsite through batteries, and the stored energy can be used to boost the power supply from the solar and wind farm once the generation starts to decline. This approach of utilizing battery intermediates as energy storage to increase the capacity factor of the electrolyzer is also considered in the literature (Hinkley et al., 2016; Kikuchi et al., 2019).

### Retailing byproducts

Additional incentive also lies in retailing the byproduct oxygen and heat generated while operating the electrolyzer system. Roughly 8 kg of O_2_ are generated per kg of H_2_ (based on simple stochiometric balance), with very high purity (>99%). We note that by retailing the by-product oxygen, we can reduce LC_H2_ by A$0.4 per kg of hydrogen generated, assuming a retail oxygen price to be A$50 t_O2_^−1^ (Finke et al., 2021). This oxygen can be retailed to consumer industries like refining operations or pharmaceutical companies or used as a fuel in metal smelters, etc. Oxygen is also used in the water treatment system, especially the recycling of wastewater, and this could be advantageous if large electrolyzer systems are installed near wastewater treatment plants where the oxygen can be supplied to the treatment facility and the treated water can be used for electrolysis. Similarly, heat is also generated during operation due to the internal resistance of the electrolyzer, which is taken out of the system with an external cooling system (in the electrolyzer BoP) to ensure that the temperature remains within the optimum limit to ensure the stability of catalyst and efficiency. This heat instead could also be taken into use downstream of the electrolyzer in combined heat and power systems or in downstream Power-to-X processes, where both renewable hydrogen and heat can be utilized to produce chemicals and fuels.

### Outlook of electrolysis in Australia

The summary of the LC_H2_ established in our analysis for the different configurations is represented in Table 3. Overall, in the near term (operations starting in 2021), the wind operated configurations have the lowest average LC_H2_ between A$4 and 9 kg^−1^, taking advantage of a higher capacity factor than solar PV-operated configurations, which enables them to offset the higher cost of wind electricity than solar electricity. On the other hand, the cost of solar operated configuration is in a higher range between A$7.1 and 10.1 kg^−1^. In comparison, by operating the electrolyzer on the grid through average wholesale pricing, we can observe LC_H2_ between A$5.6 and 7.8 kg^−1^. Operating the electrolyzer through the wholesale electricity pricing on the grid does benefit as it allows for high-capacity factor operation, where a marginal increase in electricity pricing (A$ MWh^−1^) can be traded off with higher capacity factors and lower average LC_H2_. This advantage comes at the cost of generating non-green hydrogen due to the associated CO_2_ emissions from the fossil fuel electricity generation.

**Table 3:** Summary of LC_H2_ of the 10 MW AE and PEM systems for the analyzed power supply scenario.

Scenario	AE LC_H2_ (A$ kg^−1^)		PEM LC_H2_ (A$ kg^−1^)	
	Window #1 (2021–2040)	Window #2(2031–2050)	Window #1(2021–2040)	Window #2(2031–2050)
Grid – c_*f*_: 97%	5.6–6.3	–	7.0–7.8	–
Solar PV PPA operated – c_*f*_: 31%	7.0 (4.0–10.4)	5.3 (3.3–7.2)	8.9 (5.9–12.2)	5.0 (3.2–7.6)
Off-grid PV (10 MW) – c_*f*_: 30%	7.6	5.6	10.1	5.2
Off-grid PV (Oversized) – c_*f*_: 31%	7.1	5.2	9.2	4.8
Wind PPA operated – c_*f*_: 41%	6.2 (4.1–8.6)	5.0 (3.7–6.3)	7.3 (5.3–9.9)	4.6 (3.2–6.7)
Off-grid wind (10 MW) – c_*f*_: 48%	5.6	4.8	7.2	4.3
Off-grid wind (oversized) – c_*f*_: 58%	5.4	4.6	6.7	4.2

Note: Here, the average cost for the PPA-operated configuration has been represented. The LC_H2_ of the grid was not represented for 2030 due to considerable uncertainty in Australia's future wholesale pricing. The LC_H2_ for the grid scenario represents the range of costs depending on the state's highest and lowest average electricity pricing. The LC_H2_ for the PPA scenario represents the average LC_H2_ and the LC_H2_ range depending on the lowest to highest electrolyzer capital cost (in brackets).

In comparison, it is projected that renewable electricity coupled with energy storage will contribute an increasing share of total generation (which at present is dominated by fossil fuels). Deployment of larger volumes of flexible and dynamic electrolyzers will likely facilitate and hence incentivize integration of larger volumes of variable renewable energy on to the grid. In this manner, the electrolyzer systems can act as a means of grid firming where the surplus renewables are sourced as a PPA to operate electrolyzers. In our case, however, as we consider only a 10 MW capacity system, the addition would play an insignificant role in the costs and carbon emissions of the current national electricity grid. Future work will investigate this prospect in more detail. Nevertheless, such transition to low-cost renewable-based energy is already observed in Tasmania and Queensland, where longer durations of low-cost electricity on the grid leads to LC_H2_ comparative with the lowest cost scenario of wind-operated electrolyzers. In addition, oversizing the solar and wind farms (in both stand-alone and through PPAs) would also provide a further opportunity to reduce the LC_H2_ by enhancing the availability (capacity factors) of low-cost renewable electricity. In essence, the NEM market is progressing toward a scenario where H_2_ can be generated below A$2–3 kg^−1^; however, the transition can be expedited by enhancing the availability of low-cost renewable projects both on and off the grid.

## Conclusion

Herein, we develop a techno-economic framework for developing electrolyzer projects to generate hydrogen out a techno-economic evaluation of H_2_ production from a commercial-scale 10 MW electrolyzer plant in Australia. The framework was then established to assess the costs of generating hydrogen under different electricity supply configurations of utilizing electrolyzer systems powered from the grid (fossil fuel), grid renewables, and onsite renewable generation. Based on the expected current electrolyzer capital costs (2020) and anticipated renewable electricity pricing from the Australian National Energy Market (2021–2040), long-term hydrogen supply (20-year project life using renewable electrolysis) and the cost of generating hydrogen (LC_H2_) would lie between A$4 and 12 kg^−1^. Overall, our results reveal that it is more economical to operate electrolyzer with grid-sourced wind-based electricity (A$4–9 kg^−1^), as compared to grid-supplied solar-based electricity (A$4–12 kg^−1^). Furthermore, if the same investment is conducted until 2030, we expect that the LC_H2_ range will shrink to A$3–7 kg^−1^ based on future projected electrolyzer costs and renewable electricity pricing.

We also applied the framework to assess the cost and financing benchmark required to achieve the A$2 kg^−1^ production target set by the Australian Government, at which hydrogen is expected to be viable for key business opportunities like the export of hydrogen from Australia. We reveal that to achieve costs of A$2 kg^−1^ by 2030, a combination of factors such as reduced capital costs (A$500 kW^−1^), availability of low-cost renewable energy (<A$30 MWh^−1^ at capacity factors >30%), and/or favorable financing schemes (cost of capital <3%) is required. In addition, our framework also enables us to establish the influence of factors like electrolyzer degradation rates (loss of output voltage) as this leads to reduced efficiency over time causing the levelized cost to increase (up to 5%). Moreover, reducing the indirect costs associated with electrolyzer installation will favorably improve electrolyzer economics. These factors have usually been overlooked in previous analysis and as revealed by our framework can provide a pathway for further improvement in electrolyzer economics.

Nevertheless, it is essential to note that the costs established in the analysis are just a representation of production costs; in further work, we aim to apply the framework to actual application to determine the total supply cost of generation, transportation, and utilization of hydrogen. In this way, the framework would be improved to determine the key drivers to achieve hydrogen supply costs that make its use feasible.

### Limitations of the study

The analysis is focused on finding the cost of hydrogen generation and does not include the cost consideration of transporting the generated hydrogen for its eventual use. Additionally, it is important to highlight that the model does not consider factors like degradation of renewable plant, connection cost of renewables to the electrolyzer, land costs, and grid connection fees (especially dispatch fees). These can vary significantly on a locational basis and are difficult to generalize. Besides, the model does not incorporate cost reduction due to economies of scale, as this was beyond the scope of the analysis.

## STAR★METHODS

### Key resources table

**Table:** 

REAGENT or RESOURCE	SOURCE	IDENTIFIER
**Software and algorithms**		

NEMOSIS	(Gorman et al., 2018)	http://www.ceem.unsw.edu.au/open-source-tools

**Other**		

All relevant data for modeling	This paper	Properly cited wherever applicable, available in main reference list of the manuscript

### Resource availability

#### Lead contact

Further requests and inquires for resources can be directed to the Lead Contact, Iain MacGill (i.macgill@unsw.edu. au) or to the corresponding author: Dr Rahman Daiyan (r.daiyan@unsw.edu.au)

#### Materials availability

The study did not generate any new or unique reagents.

#### Data and code availability

The authors declare that all assumed input data is detailed within the article and supplemental information, and was adopted from public resources (literature, reports etc.) that have been appropriately cited. Any extra data can be made available by the Lead Contact upon reasonable request.

### Method details

#### Electrolyzer system boundary

The electrolyzer plants in our model are assumed to be a self-contained skid unit equipped with the stack modules and the Balance of Plant (BoP) unit (Figure S1). The BoP includes the gas conditioning units for post-production treatment of hydrogen, water, and electricity feedstock conditioning units as well as all the piping and instrumentation required to operate the electrolyzer. The electrolyzer electricity supply is compared for different on-grid and off-grid settings as elaborated later. In contrast, the water supply is predominantly assumed through desalinated water, but a comparison between fresh and recycled water options is also conducted. The systems for the water supply (water processing plant) are assumed to be outside the system boundary, it is assumed that the water will be sourced at wholesale pricing. For the off-grid scenario, the solar PV and Wind farm are assumed within the electrolyzer system boundary.

#### Electrolyzer scale

For our analysis, we consider a 10 MW project scale as it is corresponding to the scale of the electrolyzer systems that are currently being explored in Australia (e.g. the Rockhampton Electrolyzer Project(ARENA, 2020) and electrolyzers in operation (e.g. the world’s largest operating electrolyzer installed as part of the F2HR project in Japan (Hiroi, 2020). Larger electrolyzer plants also seem likely to deploy multiple units in the MW size range, rather than pursuing much larger individual units. Overall, as elaborated later on we cost the electrolyzer based on unit capacity (A$ kW^-1^), in this manner the actual capacity of the system plays an arbitrary role and our findings apply to any scale capacity.

#### Electrolyzer parameters

For the Alkaline (AE) and Polymer Electrolyte Membrane (PEM) electrolyzer installed in 2020, we consider parameters of existing commercially available electrolyzers. We analyzed various commercial AE and PEM electrolyzer systems to model our electrolyzer (Table S2). After careful deliberation, we chose the AE system by ThyssenKrupp and PEM system by Siemens respectively, as their systems are state-of-the-art, proven, reliable and very flexible (10 – 100% load capacity and 10% s^-1^ dynamic load rate). This intrinsic flexibility is essential as the electrolyzers can then be quickly ramped up and ramped down in response to the variation in the electricity supply. For electrolyzer installed in 2030, we consider projected future process improvements leading to improved system efficiencies over the long run based on data provided in literature. The current and future electrolyzer parameters considered are represented in Table S6. These assumptions are further elaborated below. For our modeling, the defined costs and performance parameters include the combined system (stack and BoP).

##### PEM electrolyzer – Siemens’ Silyzer 300

The PEM model was modeled on the parameters of Siemens’ Silyzer 300 )(Siemens Energy, 2021). Data provided by Siemens reveals that the module has a nominal power requirement of 53.8 kWh kg^-1^(61%_LHV_), operates within a load limit of 10 – 100% of maximum production range, has a water requirement of 10-liter kg^-1^ of H_2_, with a total system lifetime of 20 years and a maximum capacity factor of 95%.(Priest, 2019) The Silyzer system is based on multiple 730 kW modules (Iresneusz and Zimmermann, 2019), we assume a stack based on 14 modules to build a 10 MW (~10.2 MW) system for our analysis, assuming that the same specific power consumption (53.8 kg kWh^-1^) to the overall system.

##### AE electrolyzer – Thyssenkrupp’s 10 MW module

In comparison, the AE system was modeled based on specifications of Thyssenkrupp’s 10 MW AE module which has a design capacity of 2,000 Nm^3^ hr^-1^ (~180 kg hr^-1^), that gives a power consumption of ~48 kWh kg^-1^ (68%_LHV_)(Thyssenkrupp Udhe Chlorine Engineers, 2021). The module data sheet also specifies that the water requirement of <1 liter per Nm^3^ (~11 kg hr^-1^) and load rating of 10% - 100%.

##### Literature adopted parameters

Neither of the data sheets provide an expectation of the stack lifetimes. So we consider data collected by the literature(Schmidt et al., 2017), that suggests a range of electrolyzer stack lifetimes of 40k – 80k hours (here k = 1,000) for AE and 40k to 100k hours for PEM due to high level of uncertainty in expectations. We assume that the low-end lifetime of 40k for both system as a base case and provide a comparison for higher lifetimes. In addition, analysis by Carmo et al. suggests a degradation rate of upto <3μV hr^-1^ for the AE system and <15μV hr^-1^ for PEM as expectable range under continuous operating ranges (Carmo et al., 2013). Bertuccioli et al. also suggest that the degradation rate of PEM system can be as high as ~15 μV hr^-1^ but expects the range to be <5μV hr^-1^ for state of the art system (Bertuccioli et al., 2014). In this manner, considering a 3μV hr^-1^ for AE and 5μV hr^-1^ of PEM will lead to ~1% decrease in voltage per year (assuming the state of the art cell voltage of 2.4 V for AE and 2.2 V for PEM (Carmo et al., 2013) under continuous operation. There is a limited data on the variation of degradation rates in dynamic operation, a major reason why most other analysis does not consider degradation, for simplification we consider the degradation rates of 1% year^-1^ in all analyzed cases and compare results for lower degradation rates.

For the systems installed in the 2030, in addition to cost reduction we focus on efficiency improvements as a major cost driver. There are several expectations for improvement of electrolyzer parameters, leading to an uncertain range (Schmidt et al., 2017; IEA, 2019; (IRENA, 2020a)). Schmidt el al. provide an in depth analysis of future electrolyzer parameter based on discussions from experts (Schmidt et al., 2017). They suggest a range of specific energy consumption of 42 – 61 kWh kg^-1^ AE and 45 – 61 kWh kg^-1^ for PEM system. We assume the low-end range 42 kWh kg^-1^ (85% _HHV_) for AE and 45 kWh kg^-1^(83%_HHV_) for PEM. We also provide an analysis if lower efficiency is expected.

#### Currency and inflation adjustment

We base all our calculations on Australian dollar (2020 basis); thus, all cost values (unless stated otherwise) were converted to A$ and wherever required the costs were converted based on the average exchange rates for each year as shown in Table S3. In addition, we also account for inflation by adjusting the costs using the Consumer Price Index factor (Table S4)

#### Electrolyzer capital cost

We adopted quoted/projected expected electrolyzer purchase costs from various literature resources for 2020 and 2030. Depending on the reference source (Table S3), these quoted costs vary in currency and basis year, for consistency we adjust these costs to A$2020 basis as suggested earlier (Table S5). In each year, a range of data was achieved reflecting uncertainty of costs, these costs were then arranged in ascending order by ranking the lowest to highest costs (Figure 2). The subsequent arithmetic mean of the costs in each range was then used as an average representation of electrolyzer purchase cost in that range. While the low and high purchase cost were later used in the analysis to represent the uncertainty and a probable range of hydrogen generation costs depending on the range of electrolyzer costs in each year.

In addition to the purchase costs, we consider the indirect costs of importing the electrolyzer equipment from Germany and installing it at site. These costs are assumed as a fraction of the electrolyzer’s uninstalled (purchase) cost. A major component of these costs would be associated with importing the electrolyzer, we assume the import duty to be equivalent of the Australian sales tax of 10%. In addition, we also assume the cost of freight that includes a freight cost, which would be roughly 2% of the electrolyzer purchase costs and an extra 1% to account for insurance. We also assume additional 2% of the purchase cost to cover inland transport in Australia. These assumptions were based on analyzing the freight charges and contracts of transporting similar equipment to and within Australia. To summarize, this leads to an overall electrolyzer import cost of 15% of the electrolyzer purchase cost in 2020. Note that for 2030, we assume that the installation factor cost would decrease to 10% of uninstalled cost, possibly arising from improvement in the electrolyzer supply chain especially through subsidies on the import duty provided by the government to support electrolyzer projects. Moreover, we also assume a cost of 19% of the uninstalled cost for site preparation with 9% contributing to labor costs and 10% for material costs. An additional 1% of the uninstalled cost is considered to cover the front-end engineering design (FEED) services costs and attain any upfront licensing fees. To account for contingencies, we assume a high project contingency costs of 15%, due to probable uncertainties in project financing as such projects currently would be a first of the kind in Australia. For window 2 (2030 - 2050), we assume that the contingency will decrease to 10% provided various electrolyzer projects would have been conducted, resulting in lower uncertainty in project costing.

#### Electrolyzer operating cost

The electrolyzer operating costs (OPEX) include fixed and variable operational costs. The fixed OPEX include electrolyzer operation and maintenance (O&M) cost, major stack replacement, as well as miscellaneous costs like a tax shield, interest, and ongoing licensing requirements (assumed as 2.5% of the total capital cost per year). An important operating cost is stack replacement, which is expected to be due once the electrolyzer has been utilized for cumulative hours corresponding to the stack lifetime. Analysis in literature, suggests that the cost of replacing the stack can be between 5 to 40% of the electrolyzer purchase cost. We assume a base case value of 20% of the electrolyzer purchase cost. It is assumed that the replaced stack will have a higher efficiency than the initially installed stack given that electrolyzer efficiency is expected to keep improving through R&D.

For the standalone integrated electrolyzer case, the capital and operating cost of the solar and wind farms was added to the electrolyzer. The capital and operating costs of each operational configuration are shown in Tables S9 and S10.

#### Electrolyzer partial output level

Both the electrolyzer systems can operate with dynamic loads between 10 – 100% of maximum capacity (Table S2). To represent this, we assume the operating load as the electrolyzers’ “partial output level”, i.e., the ratio of the electrolyzer’s actual operating load to the maximum load capacity (10 MW). In the grid configuration, due to the readily available electricity the electrolyzer can be operated at maximum load at all times of operation. However, for the renewable operated configuration the load will vary due to the intermittent power output of the solar and wind farm. Given that both the AE and PEM system have the minimum operating level of 10% (Table 3), and the electricity supply capacity factor were adjusted to ensure the electrolyzer output level always remains above 10% while operating.

#### Capacity factor

Here we define the capacity factor as the actual power output level of the electricity supply in a year to the maximum power output the electricity supply can generate during a year if operated throughout at maximum capacity. Moreover, for our analysis as all the power output from the electricity supply is consumed to operate the electrolyzer, the capacity factors the electrolyzer achieves will depend on the electricity supply.

#### Wholesale electricity pricing

To model the grid pricing, we consider the distribution of the wholesale regional prices over the FY2018-19 for each state region of the Australian National Electricity Market (NEM) made available by Australian Energy Market Operator (AEMO) as part of its market information system. The data by AEMO was then filtered using the opensource tool “NIMOSIS”, which is designed specifically for the Australian National Energy Market(Gorman et al., 2018). The tool is accessible on the Collaboration on Energy and Environmental Markets website.

The price duration data is collected over a 30-minute window and can then be represented through price duration curves over the 8,760 hours in a year (Figure 4A), by ordering these prices from lowest to highest. The price duration curves then provide a simple yet useful means to determining the proportion of time that prices were below or above any given price. These curves can be used in two ways:

(i) firstly, to determine the maximum ‘break-even’ electricity price to make commercially viable H_2_. The capacity factors at which this can be achieved is represented as a solid line for each NEM state in Figure 4A. This way for example, a breakeven price of A$100 MWh^-1^ would have seen an electrolyzer in the state of Victoria (VIC), SA) and Tasmania (TAS) achieve around a 60% capacity factor (i.e., operate at 100% electrolyzer capacity for over 60% of half-hour periods over the year).

(ii) secondly, a running average of the price is also provided (Figure 4B), which offers further guidance on how flexible loads can save on electricity costs. For example, an electrolyzer in South Australia that operated at a very high-capacity factor in 2019 would have paid an average A$100 MWh^-1^ over the year. If the load had only run in those hours where the price was below the median for the year (i.e., an overall 50% capacity factor) it is average electricity price over the year would be only around A$60 MWh^-1^.

#### PPA contract costs

To model the costs of solar and wind PPAs, we take real-time market data from the NEM, which shows much of the investment in wind and solar projects are currently underpinned by a range of Power Purchase Agreements (PPAs) with large energy consumers. These contracts are effectively variable volume derivative contracts with a contract price and period that are settled based on the future spot price. As such, they can effectively lock in a future A$ MWh^-1^ spot price for a load.

Given that it is economically ideal that the electrolyzer is operated at high nominal outputs (as close to 100%) as long as possible, we assume a fixed volume PPA identical to the nameplate capacity of electrolyzer (10 MW in the analyzed case) to operate the electrolyzer. Any potential surplus power generated that is not consumed by the electrolyzer can then be sold by the PPA provider onto the grid instead of curtailment. However, this would limit the electrolyzer's overall capacity factor, which would be dictated by the generation profile of the contracted solar/wind plant with which it has an agreed PPA. For Australia, the average capacity factor of currently operating solar and wind farms is 29% and 40% respectively, which are expected to increase to 31% and 46% by 2050 (Figure 2) ((Aurecon; Australian Energy Market Operator AEMO, 2019).

The cost of the PPA (A$ MWh^-1^) was then assumed as the levelized cost of electricity (LCOE) from the contracted solar and wind farms. These LCOE (A$ MWh^-1^) were evaluated by assuming a simple annuity cost of capital cost of electrolyzer (calculated by annualizing the build cost of the solar/wind farm through a weighted average cost of capital – WACC) plus any additional operating cost of the plant and dividing these by the capacity factor of the plant. As shown in Equation 1:

(Equation 1) $$LCOE\left(\frac{\text{A\$}}{\text{MWh}}\right)=\;\frac{{\text{C}}_{\text{R}}\times \text{ CAPEX}+\text{OPEX}}{\text{MW}.c_{f}.8760\text{ hrs }}$$

Here the C_*R*_ factor is the capital recovery factor, which is elaborated later in Equation 4. The build costs (CAPEX) of solar and wind farms were then adopted from the data provided by Australian Energy Market Operator (AEMO), that has suggested the costs of developing grid-connected solar and wind farm up to 2050 under different scenarios of energy mixes (Table S7) (Graham et al., 2020). Given that the cost of building solar and wind farm is decreasing and the capacity factor increasing year on year, the LCOEs are expected to decrease, and we consider that cheaper PPAs would be available as new systems come online (Table S8). We do not consider the cost of transmission or any supply connection costs.

#### Solar PV and wind farm duration curves

For the off grid standalone configuration, the electrolyzer is assumed to be operated with dedicated solar and wind farm thus the electrolyzer capacity factor is limited by the duration profile of the solar and wind farm. To model the generation profile of these hypothetical plants for our electrolyzer, we considered generation profile of existing power plants in the NEM. The generation data for the NEM connected solar and wind power plants are provided also by AEMO and can be filtered using NIMOSIS. We used 30-minute generation data from FY2018-19 and ordered from highest to lowest 30-minute period operation over a year to develop a duration curve. These duration curve then would corelate with the electrolyzer’s partial output level during operation (Figures 7 and S12). In this manner, the average electrolyzer power output across the whole year would represent the capacity factor of the electrolyzer. Moreover, the area under these duration curves would represent the actual available energy each year that the electrolyzer can convert to hydrogen.

##### Similar capacity renewable generator

The duration curve reveals that a similar capacity (10 MW) utility PV plants (Figure S10) would only operate the electrolyzer for only 40% of hours over the year. In contrast, a similar capacity wind plant (Figure S10) might operate the electrolyzer at least some level of output for over 80% of the year. To represent this, we evaluate the renewable generators’ "capacity factor” i.e., the actual power output – MWh (area under the duration curve) to the maximum power output of the renewable generator (power generated if the electrolyzer runs at full capacity throughout the year). The duration curve will vary based on the performance of each solar or wind farm, we consider different solar and wind farms across Australia for the analysis. In addition, given that the electrolyzer has the lowest load rating of 10% of maximum capacity we assume that the solar and wind farm is curtailed to match this load limitation.

##### Oversized/undersized renewable generator

The generation duration curves also provide a simple yet useful method for exploring oversizing potential. For instance, a larger capacity solar (14 MW) or wind (12.5MW), could potentially operate the electrolyzer at any given power rating for a longer duration (Figure S12). To be specific, the electrolyzer would now operate at the maximum rated capacity (10 MW) for over 25% of the year, and at partial capacity (< 10 MW) for an additional 20% of the year. Similarly, by oversizing the wind generator so that the electrolyzer is at 80% of the Wind plant capacity (i.e., a 10 MW electrolyzer is operated with a 12.5 MW Wind plant), we can operate the electrolyzer at maximum capacity for 30% of the year and at partial capacity for another 40% of the year. We analyze different oversizing scenarios using different “*size factors*” (i.e., the ratio of the solar/wind farm’s nameplate capacity to the electrolyzer system nameplate capacity) and data from various solar/wind farms across Australia to determine the optimal operation configuration.

#### Levelized cost of hydrogen - LCH2

The cost of generating hydrogen was calculated by determining the capital and operating costs of the electrolyzer system as well as the amount of hydrogen generated. The levelized costs were calculated by levelizing the discounted capital and operating costs for the total project life over the discounted amount of hydrogen produced over the lifetime. This gives the levelized cost of hydrogen – LC_H2_ as shown in Equation 2. In the equation, C_R_ represents the capital recovery factor that converts the total capital requirement (CAPEX) into a present value annuity costs representing the yearly cost to recover the capital invested. This annualized capital cost is referred to as capital recovery in the rest of the analysis. P_R_ is the hydrogen production rate, and O_C_ is the annual operating cost. The production rate is determined by correlating the energy available (MWh) to the electrolyzer in any given year and the electrolyzer's specific energy consumption (SEC – kg kWh^-1^).

(Equation 2) $$LC_{H2}\left(\frac{\text{A\$}}{{\text{kg}}_{\text{H}2}}\right)=\;\frac{{\text{C}}_{\text{R}}\times \text{ CAPEX}+{\text{O}}_{\text{C}}}{{\text{P}}_{\text{R}}.{\text{ c}}_{\text{f}}.8760\text{ hrs }}$$

This equation reflects how the capacity factor becomes a key consideration, as it dictates the amount of energy from available to the electrolyzer in the whole year that can be converted to hydrogen. For the PPA operated scenario, we evaluated the LC_H2_ for each individual year (Figure 4) depending on the cash flows in each year i.e., the capital recovery, operating costs, and cost of stack replacement. This allows for a simplistic representation of the effect of the electrolyzer degradation and efficiency. In the off-grid case, the additional capital and operating costs of the Solar PV and Wind Farm were added to the costs of the electrolyzers.

In addition, it is also important to note that this LC_H2_ reflects the cost of generating H_2_ and does not include the cost of supply that would have to incorporate the additional cost of storage, compression, and transmission. These costs would be region and application specific, and as a simplification these costs have not been incorporated, however the model can be adapted to analyze specific cases by incorporating the additional capital and operating costs.

#### Capital recovery factor

The capital recovery factor (C_R_), in turn, is dependent on the weighted average cost of capital (WACC), as represented by Equation 3. In the equation, n is assumed as the project life is considered 20 years for the electrolyzer as BoP and electronic systems are designed for a long lifespan. Such lifetimes are expected provided periodic maintenance and timely electrolyzer stack replacement are undertaken.(Colella et al., 2014) In addition we assume that the life of the solar and wind farm is 25 years, which is standard for renewable projects in Australia ((Aurecon; Australian Energy Market Operator AEMO, 2019). This reflects a mismatch, as the solar PV/Wind farm can be operated for an additional useful life of 5 years compared to the electrolyzer. This is of particular importance in the off-grid case, given that we assume the electrolyzer project would be after 20 years, the additional life of the solar PV/Wind farm (5 years) can be recovered by retailing the generatable electricity to the grid or local consumer. The summary of the assumed parameters for the LC_H2_ and WACC are presented in Table 2.

(Equation 3) $$C_{R}=\;\frac{{\text{WACC }\left(1+\text{WACC}\right)}^{\text{n}}}{{\left(1+\text{WACC}\right)}^{\text{n}}-1}$$

#### Weighted average cost of capital - WACC

The capital recovery in turn depends on the weighted average cost of capital (WACC). The WACC in tun depends on the share of debt and equity and the subsequent rate of return on capital that would be required to recover the capital investment. The WACC is readily used to measure the cost of capital for renewable energy projects, and various WACC’s have been defined to represent different financing options for the capital costs (Steffen, 2020). For this analysis, we consider the “vanilla WACC” that assumes a weighted average cost of capital based on the weighted average of pretax equity and debt-based investment, as shown in Equation 4:

(Equation 4) WACC=Share of Equity ×Cost of Equity+Share of Debt × Cost of Debt

We assume that the same WACC would apply to the solar PV, wind farm and electrolyzers as they can all be categorized as renewable projects. AEMO also provides a periodic outlook for the financing breakdowns of renewable projects in Australia. Their recent report shows that most renewable energy projects capital costs are financed with a split of 25% debt and 75% equity(GDH; AEMO, 2018). While the cost of debt ranged between 4.5 – 5.5% (average: 5%) and the cost of equity range between 7 – 12% (average: 10%). We assume that the same WACC would apply to the solar PV, wind farm and electrolyzers. Given these assumptions, the WACC was evaluated as 6.25% (for average cost of debt and equity assumptions). The WACC is a critical factor as it defines the capital recovery which is a major cost driver. As noted above, our economic analysis seeks to better capture the real costs of project implementation including financing and other indirect costs than many of the existing studies in the academic literature.

### Quantification and statistical analysis

We used the arithmetic mean as a quantitative representation of the purchase and capital cost of the electrolyzer. In each year, a range of data was achieved reflecting uncertainty of costs, these costs were then arranged in ascending order by ranking the lowest to highest costs (Figure 2). The subsequent arithmetic mean of the costs in each range was then used as an average representation of electrolyzer purchase cost in that range. While the low and high purchase cost were later used in the analysis to represent the uncertainty and a probable range of hydrogen generation costs depending on the range of electrolyzer costs in each year. This description has been added to Table 1, title of Figure 2 and footnote of Table S5.

## Supplemental information

Document S1. Figures S1–S13 and tables S2–S10

Table S1. Detailed comparison of frameworks in literature for assessing hydrogen production costs using electrolyzer with our proposed framework and model All values have been converted to A$ basis based on exchange rates of 1 USD$ = 1.29 A$ and 1€ = 1.54 A$ (the costs were not adjusted for reference year). For the Considerations column: N.C. represents Not Considered, Y represents Considered, and U.S. represents Unspecified. LC_H2_ represents the levelized costs of hydrogen. The comparison was used to highlight the research gap and justify our analysis in the main text and STAR Methods.

## Footnotes

Supplemental information can be found online at https://doi.org/10.1016/j.isci.2021.102539.
