# Supplementary material for: Designing optimal integrated electricity supply configurations for renewable hydrogen generation in Australia
Source: iScience. 2021 May 15;24(6):102539. doi: 10.1016/j.isci.2021.102539 (PMC8184509; doi:10.1016/j.isci.2021.102539)
Supplement: Document S1. Figures S1–S13 and tables S2–S10 [file mmc1.pdf]

iScience, Volume *24*

Supplemental information

Designing optimal integrated electricity supply configurations for renewable hydrogen generation in Australia

Muhammad Haider Ali Khan, Rahman Daiyan, Zhaojun Han, Martin Hablutzel, Nawshad Haque, Rose Amal, and Iain MacGill

Table S2. List of some commercially available AE and PEM electrolyzers worldwide. Related to STAR Methods

**Manufacturer** *(Origin)*	**Technology**	**Capacity** (kW/MW)	**Production Rate** ($Nm^3 hr^{-1}$)	**Water Consumption** ($l\ Nm^{-3}$ of H_2)	**Power Consumption** ($kWh\ Nm^{-3}$ of H_2)
Acta *(Italy)* (Acta S.p.A., 2019)	AE	4.7 kW	1.00	0.80	4.20
Pure Energy Center *(United Kingdom)* (Pure Energy Centre, no date)	AE	213 kW	42.62	0.84	4.90
Hydrogenics *(USA)* (Hydrogenics, no date)	AE	384 kW	60.00	1.00	5.20
ErreDue S.P.A *(Italy)* (ErreDue SPA, no date)	AE	960 kW	170	0.82	5.36
McPhy Energy *(France)*(McPhy, 2018)	AE	4 MW	400	NA	4.50
NEL *(Norway)*(NEL Hydrogen, 2020)	AE	N.A.	3,880	0.90	4.40
Green Hydrogen *(Denmark)* (Green Hydrogen, no date)	AE	277 kW	90	1.00	3.07
Idroenergy *(Italy)* (Idroenergy, no date)	AE	448 kW	80	0.80	5.60
iGas Energy (Germany)(iGas Energy, no date)	PEM	2 MW	320	1.00	5.30
NEL *(Norway)* (Nel Hydrogen, no date)	PEM	2 MW	413	0.90	4.53
H-Tec Systems *(Germany)* (H-Tec Systems, no date)	PEM	1.4 MW	210	1.60	5.00
H2B2 *(Spain)* (H2B2 Systems, no date)	PEM	2.1 MW	414	1.00	5.00

Table S3. Reported purchase cost of electrolyzer in literature. Related to Table 1, S4 and S5, and STAR Methods

**Source** – Currency Basis	AE		PEM	
	2020	2030	2020	2030
**Bertucciolli** *et al.* – €$_{2014}$ kW^{-1}	370 – 900 (630)	370 – 800 (580)	370 – 1,300 (1,000)	250 – 1,270 (760)
**Glenk** ***et al.*** – €$_{2016}$ kW^{-1}	550 – 1,000	500 – 700	1,200 – 1,600	500 – 900
**Schmidt** ***et al.*** – €$_{2016}$ kW^{-1}	700 – 1,400 (1,300)	700 – 1,000 (750)	1,000 – 1,400 (1,300)	700 – 1,980
**Saba** ***et al*.** – €$_{2017}$ kW^{-1}	685 – 1,388 (877)	600 – 906 (787)	1,373	676
**IEA** – U\$$_{2019}$ kW^{-1}	500	400	1,100	654
**IRENA** – U\$$_{2019}$ kW^{-1}	840	200	–	–
**BNEF** – U\$$_{2019}$ kW^{-1}	1,200	115 – 135	1,400	425 – 1,000
**OEM Quoted** – €$_{2020}$ kW^{-1}	–	–	1,000	500

Note: The electrolyzer purchase costs are collected from various literature and analysis reports. The OEM quoted prices were obtained through correspondences with commercial electrolyzer manufacturers. The costs are quoted in various currencies and were updated through the exchange rates and adjusted to present values through Consumer Price Index (CPI) in Australia. These exchange rates and CPI index are presented in **Table S4**.

Table S4. Annual Exchange rates and Consumer Price Index in Australia. Related to Table S5 and STAR Methods

Year	U$: A$	€: A$	CPI	CPI Correction Factor
2014	1.22	1.47	106.6	1.09
2015	1.37	1.48	108.4	1.07
2016	1.39	1.49	110	1.06
2017	1.28	1.47	112.1	1.04
2018	1.43	1.58	114.1	1.02
2019	1.43	1.61	116.2	1.00
2020	1.35	1.66	116.2	1.00

Note: The CPI correction factor reflects the adjustment in costs required to attain present value costs. The factor is calculated as the CPI index in the base year (2020) to the CPI index each year. E.g., the CPI index in 2014 is 106.6 and that in 2020 is 116.2 so the CPI correction factor is equal to 116.2/106.6 = 1.09

Table S5. Electrolyzer purchase cost data used in calculating average purchase cost. Related to Table S3, S4, Table 1 & 2 and Figure 2.

**Electrolyzer System**	**AE** (A$ kW^{-1})		**PEM** (A$ kW^{-1})	
**Year**	**2020**	**2030**	**2020**	**2030**
Electrolyzer Purchase Cost from Literature (Based on **Table S2**)	A$594.02 (Bertuccioli *et al.*, 2014)	A$540.54 (IEA, 2019)	A$1,123.82 (Bertuccioli *et al.*, 2014)	A$401.36 (Bertuccioli *et al.*, 2014)
	A$675.68 (IEA, 2019)	A$594.02 (Bertuccioli *et al.*, 2014)	A$1,485.00 (IEA, 2019)	A$574.32 (BNEF, 2019)
	A$865.04 (Bertuccioli *et al.*, 2014a)	A$786.40 (Glenk and Reichelstein, 2019)	A$1,572.80 (Schmidt *et al.*, 2017)	A$675.68 (Suggested by Manufacture)
	A$1,011.44 (Saba *et al.*, 2017)	A$931.16 (Bertuccioli *et al.*, 2014)	A$1,605.45 (Bertuccioli *et al.*, 2014)	A$786.40 (Glenk and Reichelstein, 2019)
	A$1,077.36 (Glenk and Reichelstein, 2019)	A$943.68 (Saba *et al.*, 2017)	A$1,659.39 (Suggested by Manufacture)	A$882.90 (IEA, 2019)
	A$1,108.83 (IRENA, 2019)	A$1,100.96 (Schmidt *et al.*, 2017)	A$1,887.35 (Glenk and Reichelstein, 2019)	A$965.71 (Saba *et al.*, 2017)
	A$1,135.14 (Schmidt *et al.*, 2017)	A$1,108.83 (Schmidt *et al.*, 2017)	A$1,891.89 (BNEF, 2019)	A$1,100.96 (Schmidt *et al.*, 2017)
	A$1,379.34 (Saba *et al.*, 2017)	A$1,188.03 (Saba *et al.*, 2017)	A$1,958.57 (Saba *et al.*, 2017)	A$1,220.14 (Bertuccioli *et al.*, 2014)
	A$1,444.91 (Bertuccioli *et al.*, 2014)	A$1,237.79 (Glenk and Reichelstein, 2019)	A$2,044.63 (Schmidt *et al.*, 2017)	A$1,351.35 (BNEF, 2019)
	A$1,572.80 (Schmidt *et al.*, 2017)	A$1,284.36 (Bertuccioli *et al.*, 2014)	A$2,087.09 (Bertuccioli *et al.*, 2014)	A$1,415.52 (Glenk and Reichelstein, 2019)
	A$1,621.62 (BNEF, 2019)	A$1,424.95 (Saba *et al.*, 2017)	A$2,516.47 (Glenk and Reichelstein, 2019)	A$2,038.93 (Bertuccioli *et al.*, 2014)
	A$2,059.25 (Glenk and Reichelstein, 2019)	A$1,584.04 (Schmidt *et al.*, 2017)		
	A$2,183.04 (Saba *et al.*, 2017)			
	A$2,217.66 (Schmidt *et al.*, 2017)			
**Average** (A$ kW^{-1})	A$1,353.29	A$1,802.95	A$1,060.40	A$1,037.57
**Lowest** (A$ kW^{-1})	A$594.02	A$540.54	A$1,123.82	A$401.36
**Highest** (A$ kW^{-1})	A$2,217.66	A$1,584.04	A$2,516.47	A$2,038.93

Note: Here the average cost represents the arithmetic mean of all the purchase costs in each range (AE – 2020/2030 and PEM – 2020/2030). While the lowest and highest costs were considered as measure of uncertainty, refer to **STAR Methods**.

Table S6. Technical Specifications of AE and PEM Electrolyzer System in this study. These values were used to model the AE and PEM system in each configuration. **Related to STAR Methods and Table 1**.

**Parameter**	**10 MW AE System**	**10 MW PEM System**
Specific Electricity Consumption - SEC	48.3 kWh kg^{-1} of H_2 (2020) (ThyssenKrupp Uhde Chlorine Engineers, 2018) 43 kWh kg^{-1} of H_2 (2030) (Bertuccioli *et al.*, 2014; Schmidt *et al.*, 2017)	53.8 kWh kg^{-1} of H_2 (2020) (Siemens, 2018; Priest, 2019) 45 kWh kg^{-1} of H_2 (2030) (Bertuccioli *et al.*, 2014; Schmidt *et al.*, 2017)
Electrolyzer Efficiency (HHV%)	>82% (2020) 85% (2030) (Bertuccioli *et al.*, 2014; Schmidt *et al.*, 2017)	>75% (2020) 83% (2030) (Bertuccioli *et al.*, 2014; Schmidt *et al.*, 2017)
Specific Water Consumption	1 L Nm^{-3} of H_2 (ThyssenKrupp Uhde Chlorine Engineers, 2018) 11 L kg_{H2}^{-1} (1 Nm^3 of H_2 = 0.089 kg of H_2)	~1 L Nm^{-3} of H_2 (Siemens, 2018; Priest, 2019) 10 L kg_{H2}^{-1} (1 Nm^3 of H_2 = 0.089 kg of H_2)
Nominal Stack Life	40k – 80k hours (Schmidt et al., 2017)	40k – 100k hours (Schmidt *et al.*, 2017)
Dynamic Load Operating Range	10 – 100% (ThyssenKrupp Uhde Chlorine Engineers, 2018)	10 – 100% (Siemens, 2018; Priest, 2019)
Load Flexibility	$\geq$ 10% sec^{-1} (ThyssenKrupp Uhde Chlorine Engineers, 2018)	$\geq$ 10% sec^{-1} (Siemens, 2018; Priest, 2019)
Electrolyzer System Degradation (Max)	1% loss in voltage per year (Refer to Methods)	1% loss in voltage per year (Refer to Methods)

Table S7. Projected capital cost of solar PV and wind farms in Australia under the Central Policy and High Variable Renewable Energy Scenario (Aurecon, 2019; Graham *et al.*, 2020). Related to Figure 2 and Table S8.

Construction Year	Central Energy Policy Scenario			High Variable Renewable Energy Scenario		
	Utility PV (A$ kW^{-1})	***Utility Wind*** (A$ kW^{-1})	***Capacity Factor*** (%)	***Utility PV*** (A$ kW^{-1})	***Utility Wind*** (A$ kW^{-1})	***Capacity Factor*** (%)
2020 – 21	1,285.00	1,884.00	29.3	1,289.00	1,891.00	40.6
2021 – 22	1,150.00	1,868.00	29.5	1,176.00	1,877.00	40.9
2022 – 23	1,060.00	1,861.00	29.6	1,100.00	1,867.00	41.2
2023 – 24	1,019.00	1,854.00	29.8	1,018.00	1,859.00	41.5
2024 – 25	984.00	1,848.00	29.9	942.00	1,851.00	41.8
2025 – 26	954.00	1,841.00	30.1	898.00	1,845.00	42.1
2026 – 27	926.00	1,836.00	30.2	869.00	1,839.00	42.4
2027 – 28	901.00	1,829.00	30.4	843.00	1,834.00	42.7
2028 – 29	878.00	1,822.00	30.5	819.00	1,827.00	43.0
2029 – 30	854.00	1,815.00	30.7	799.00	1,820.00	43.3
2030 – 31	829.00	1,809.00	30.8	779.00	1,812.00	43.6
2031 – 32	808.00	1,805.00	31.0	765.00	1,804.00	43.9
2032 – 33	788.00	1,802.00	31.0	755.00	1,796.00	44.2
2033 – 34	771.00	1,795.00	31.0	745.00	1,787.00	44.5
2034 – 35	754.00	1,788.00	31.0	721.00	1,779.00	44.8
2035 – 36	731.00	1,778.00	31.0	698.00	1,770.00	45.1
2036 – 37	712.00	1,772.00	31.0	655.00	1,763.00	45.4
2037 – 38	693.00	1,766.00	31.0	647.00	1,756.00	45.7
2038 – 39	684.00	1,763.00	31.0	620.00	1,750.00	46.0
2039 – 40	673.00	1,760.00	31.0	606.00	1,744.00	46.0
2040 – 41	665.00	1,754.00	31.0	590.00	1,731.00	46.0
2041 – 42	658.00	1,748.00	31.0	582.00	1,717.00	46.0
2042 – 43	647.00	1,742.00	31.0	574.00	1,702.00	46.0
2043 – 44	634.00	1,740.00	31.0	564.00	1,694.00	46.0
2044 – 45	617.00	1,737.00	31.0	557.00	1,687.00	46.0
2045 – 46	601.00	1,734.00	31.0	548.00	1,680.00	46.0
2046 – 47	588.00	1,731.00	31.0	541.00	1,674.00	46.0
2047 – 48	578.00	1,729.00	31.0	533.00	1,669.00	46.0
2048 – 49	570.00	1,726.00	31.0	527.00	1,664.00	46.0
2049 – 50	563.00	1,723.00	31.0	518.00	1,658.00	46.0
2050 – 51	559.00	1,721.00	31.0	513.00	1,656.00	46.0

Note: These costs are exclusive of transmission and connection costs as well as land costs (Refer to **STAR Methods**).

Table S8. Projected Levelized Cost of Electricity (LCOE) for the PPAs based on the estimated costs of Utility Solar PV and Wind Farm as a function of different WACCs (3 – 7%) for the HVRE scenario. Related to Table S6, Figure 2 and STAR Methods

Year	**Utility Wind Farm PPA** (A$ MWh^{-1})						**Utility Solar PV Farm PPA** (A$ MWh^{-1})					
**WACC (%)**	**3%**	**4%**	**5%**	**6%**	**6.25%**	**7%**	**3%**	**4%**	**5%**	**6%**	**6.25%**	**7%**
2020 – 21	39.39	42.89	46.58	50.45	**51.44**	54.48	35.46	38.77	42.26	45.91	**46.85**	49.72
2021 – 22	38.90	42.35	45.98	49.79	**50.77**	53.77	32.77	35.77	38.93	42.25	**43.10**	45.71
2022 – 23	38.48	41.88	45.47	49.23	**50.20**	53.16	30.92	33.71	36.66	39.74	**40.53**	42.96
2023 – 24	38.09	41.46	45.01	48.73	**49.68**	52.60	28.96	31.53	34.24	37.08	**37.81**	40.04
2024 – 25	37.71	41.04	44.55	48.22	**49.17**	52.06	27.14	29.51	32.01	34.62	**35.30**	37.35
2025 – 26	37.37	40.66	44.13	47.77	**48.71**	51.57	26.05	28.29	30.66	33.14	**33.78**	35.73
2026 – 27	37.03	40.29	43.73	47.33	**48.25**	51.08	25.29	27.45	29.73	32.12	**32.74**	34.61
2027 – 28	36.71	39.94	43.34	46.91	**47.83**	50.63	24.60	26.69	28.89	31.20	**31.79**	33.60
2028 – 29	36.37	39.56	42.93	46.46	**47.36**	50.13	23.97	25.98	28.11	30.34	**30.91**	32.67
2029 – 30	36.03	39.19	42.52	46.01	**46.90**	49.65	23.42	25.38	27.45	29.61	**30.17**	31.87
2030 – 31	35.68	38.80	42.10	45.55	**46.43**	49.14	22.88	24.78	26.79	28.89	**29.43**	31.08
2031 – 32	35.33	38.42	41.68	45.09	**45.97**	48.65	22.47	24.33	26.29	28.34	**28.87**	30.48
2032 – 33	34.99	38.05	41.27	44.64	**45.51**	48.16	22.23	24.06	25.99	28.01	**28.53**	30.12
2033 – 34	34.64	37.66	40.84	44.18	**45.03**	47.65	22.01	23.82	25.73	27.72	**28.23**	29.80
2034 – 35	34.31	37.30	40.44	43.74	**44.59**	47.18	21.51	23.26	25.10	27.03	**27.53**	29.04
2035 – 36	33.97	36.92	40.03	43.29	**44.13**	46.69	21.02	22.71	24.50	26.37	**26.85**	28.32
2036 – 37	33.66	36.58	39.66	42.88	**43.71**	46.25	20.11	21.70	23.37	25.13	**25.58**	26.96
2037 – 38	33.36	36.25	39.29	42.48	**43.30**	45.81	19.94	21.51	23.16	24.90	**25.34**	26.70
2038 – 39	33.07	35.93	38.95	42.11	**42.92**	45.40	19.37	20.87	22.46	24.12	**24.55**	25.85
2039 – 40	32.99	35.84	38.84	41.99	**42.80**	45.27	19.08	20.54	22.09	23.72	**24.13**	25.41
2040 – 41	32.80	35.63	38.61	41.74	**42.54**	45.00	18.74	20.17	21.68	23.26	**23.66**	24.90
2041 – 42	32.60	35.41	38.37	41.47	**42.26**	44.70	18.57	19.98	21.47	23.03	**23.43**	24.65
2042 – 43	32.39	35.17	38.10	41.18	**41.96**	44.38	18.40	19.79	21.26	22.80	**23.19**	24.40
2043 – 44	32.28	35.04	37.96	41.02	**41.81**	44.21	18.19	19.55	21.00	22.51	**22.89**	24.08
2044 – 45	32.18	34.93	37.84	40.88	**41.67**	44.06	18.04	19.39	20.81	22.31	**22.69**	23.86
2045 – 46	32.08	34.82	37.72	40.75	**41.53**	43.91	17.85	19.18	20.58	22.05	**22.42**	23.58
2046 – 47	31.99	34.73	37.61	40.63	**41.41**	43.78	17.70	19.01	20.40	21.84	**22.22**	23.36
2047 – 48	31.92	34.65	37.52	40.54	**41.31**	43.68	17.53	18.82	20.19	21.61	**21.98**	23.10
2048 – 49	31.85	34.57	37.43	40.44	**41.21**	43.57	17.40	18.68	20.03	21.44	**21.80**	22.91
2049 – 50	31.76	34.47	37.33	40.32	**41.09**	43.44	17.21	18.47	19.79	21.18	**21.54**	22.63
2050 – 51	31.74	34.44	37.29	40.28	**41.05**	43.40	17.11	18.35	19.66	21.04	**21.39**	22.47

Note: The LCOE were calculated under the capital cost assumptions (solar PV and Wind) for the HVRE scenario and increasing capacity factors from **Table S7.** The fixed operating cost of A$17 kW^{-1} for Solar PV and A$21.9 kW^{-1} for Wind farm was assumed, while the wind farm variable operating cost was estimated at A$2 MWh^{-1}. The operational life of both solar and wind farm was considered to be 25 years.(GDH, 2018; Aurecon, 2019; Graham *et al.*, 2020). The highlighted values represent the LCOE under the base WACC assumption of 6.25% (refer to **STAR Methods**)

Table S9. CAPEX breakdown of the grid, off-grid PV and off-grid wind-powered configurations. These costs are represented for plants built in 2020 and are all quoted in A$ million. Related to Figure S2.

CAPEX Parameter	Grid (10 MW)		Off-Grid PV (10 MW)		Off-Grid Wind (10 MW)		Off-Grid PV (15 MW)		Off-Grid Wind (15 MW)	
	**AE**	**PEM**	**AE**	**PEM**	**AE**	**PEM**	**AE**	**PEM**	**AE**	**PEM**
Electrolyzer Cost	13.5	18.0	13.5	18.0	13.5	18.0	13.5	18.0	13.5	18.0
Renewable Generator Cost	-	-	12.9	12.9	18.8	18.8	19.3	19.3	28.3	28.3
Installation Factor	2.0	2.7	1.29	2.05	2.0	2.7	1.29	2.05	2.0	2.7
Site Preparation Cost	2.6	3.4	0.64	1.02	2.6	3.4	0.64	1.02	2.6	3.4
Engineering/Licensing Cost	0.1	0.1	0.64	1.02	0.1	0.1	0.64	1.02	0.1	0.1
Contingencies	2.0	2.7	1.93	3.07	2.0	2.7	1.93	3.07	2.0	2.7
**Total CAPEX** (Million A$)	**20.3**	**27.0**	**33.1**	**39.1**	**39.7**	**45.9**	**39.6**	**46.3**	**48.6**	**55.3**

Table S10. OPEX breakdown of the grid, off-grid PV, and off-grid wind-powered configurations. These costs are represented for plants built in 2020 and are all quoted in A$ million. Related to STAR Methods.

OPEX Parameter	Grid (c_f = 97%)		Grid PV (c_f = 30%)		Grid Wind (c_f = 41%)		Off Grid PV (10 MW) (c_f = 30%)		Off Grid Wind (10 MW) (c_f = 41%)		Off-Grid PV (15 MW) (c_f = 36%)		Off-Grid Wind (15 MW) (c_f = 47%)	
	**AE**	**PEM**	**AE**	**PEM**	**AE**	**PEM**	**AE**	**PEM**	**AE**	**PEM**	**AE**	**PEM**	**AE**	**PEM**
Electricity Feedstock Cost	7.2	7.2	1.1	1.1	1.8	1.8	-	-	-	-				
Water Feedstock Cost	0.1	0.1	0.03	0.02	0.04	0.03	0.03	0.02	0.05	0.04	0.04	0.03	0.06	0.05
O&M Costs	0.5	0.6	0.5	0.6	0.5	0.6	0.5	0.6	0.5	0.6	0.5	0.6	0.5	0.6
Miscellaneous Costs	0.5	0.6	0.5	0.6	0.5	0.6	0.5	0.6	0.5	0.6	0.5	0.6	0.5	0.6
Fixed OPEX PV Sys.	-	-	-	-	-	-	0.17	0.17	-	-	0.27	0.27	-	-
Fixed OPEX Wind Sys.	-	-	-	-	-	-	-	-	0.22	0.22	-	-	0.25	0.25
Variable OPEX PV Sys.	-	-	-	-	-	-	-	-	-	-	-	-	-	-
Variable OPEX Wind Sys.	-	-	-	-	-	-	-	-	0.11	0.11	-	-	0.14	0.14
**Total OPEX** (Million A$/year)	**8.30**	**8.50**	**2.13**	**2.23**	**2.84**	**3.03**	**1.20**	**1.39**	**1.38**	**1.57**	**1.31**	**1.50**	**1.45**	**1.64**
**Stack Replacement** (Million A$)	4.1	5.4	4.1	5.4	4.1	5.4	4.1	5.4	4.1	5.4	4.1	5.4	4.1	5.4

Note: The electricity feedstock for the grid operated case is represented at the average cost of the NEM (**Figure S4**). Besides to account for the electricity supply cost for the off-grid system, the capital and operating costs of the Solar PV and Wind Farms are added to the total capital and operating cost of the electrolyzer facility (refer to **STAR Methods**).

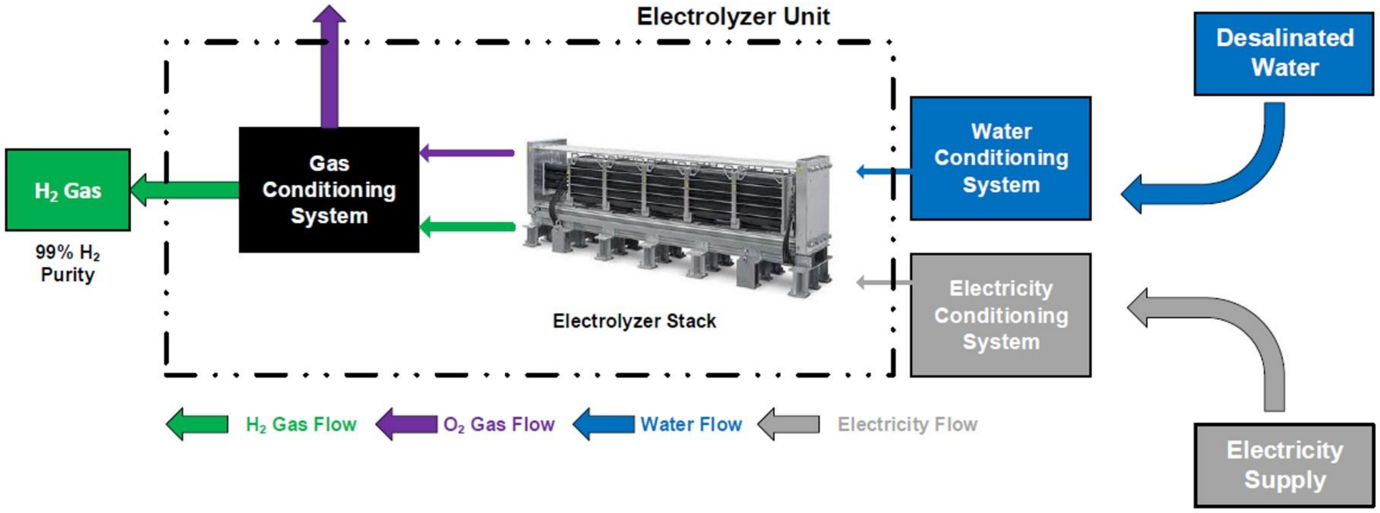

Figure S1. Simple flow scheme of the electrolyzer system showing the flow of the feedstocks and the products. The dotted line represents the boundary of the electrolyzer system. Related to STAR Methods

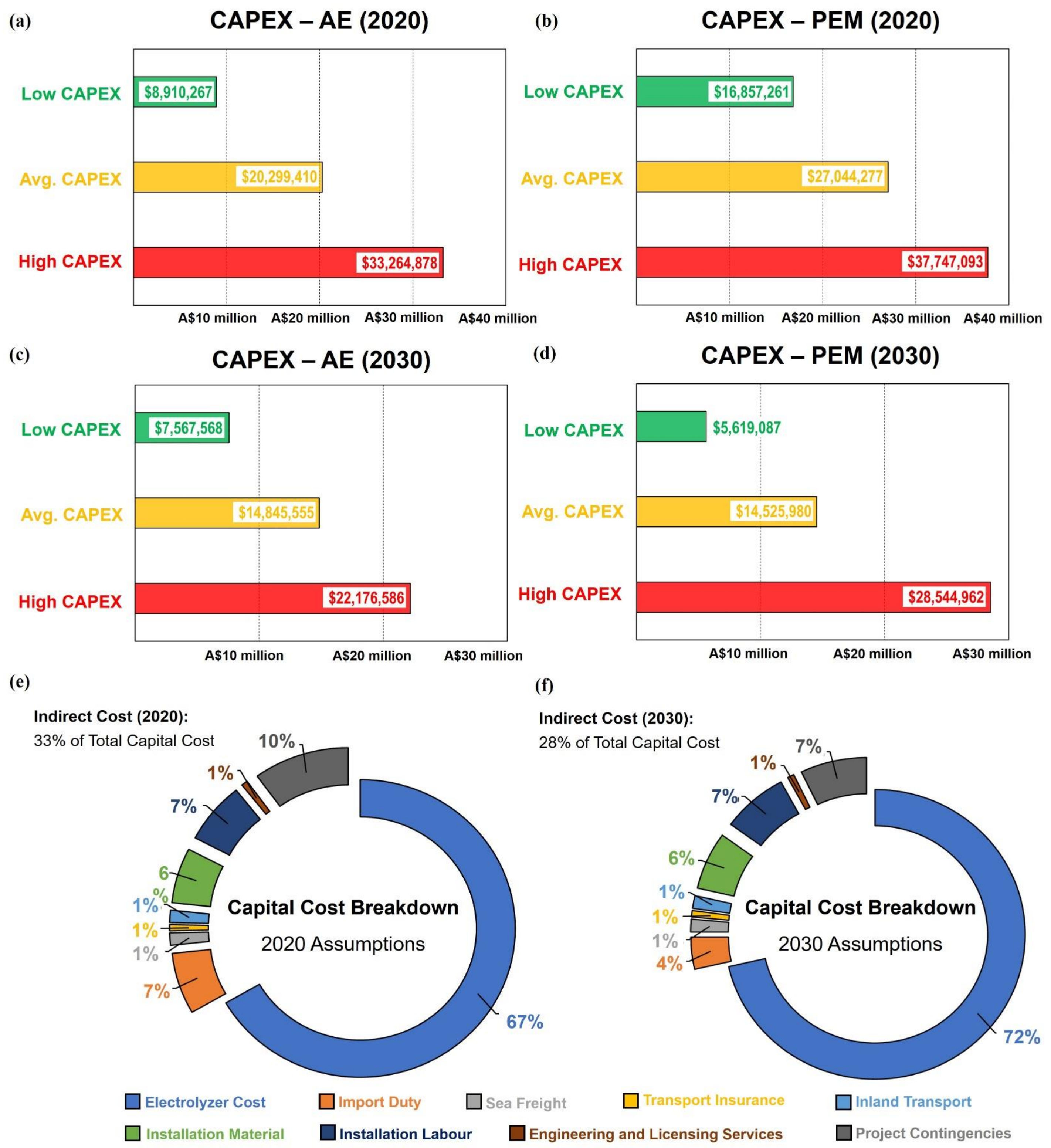

Figure S2. Estimated capital cost (CAPEX) of AE and PEM electrolyzer systems in (a-b) 2020 and (c-d) 2030. These costs are based on the electrolyzer's purchase cost (direct cost), and the modelled indirect costs including installation, facility preparation and contingency costs. (e-f) Breakdown of direct and indirect cost for 2020 and 2030. In 2020, the indirect costs were expected to have a 33% share of the total capital costs reduced to a 28% share by 2030**. Related to STAR Methods, main text, and Table S9.**

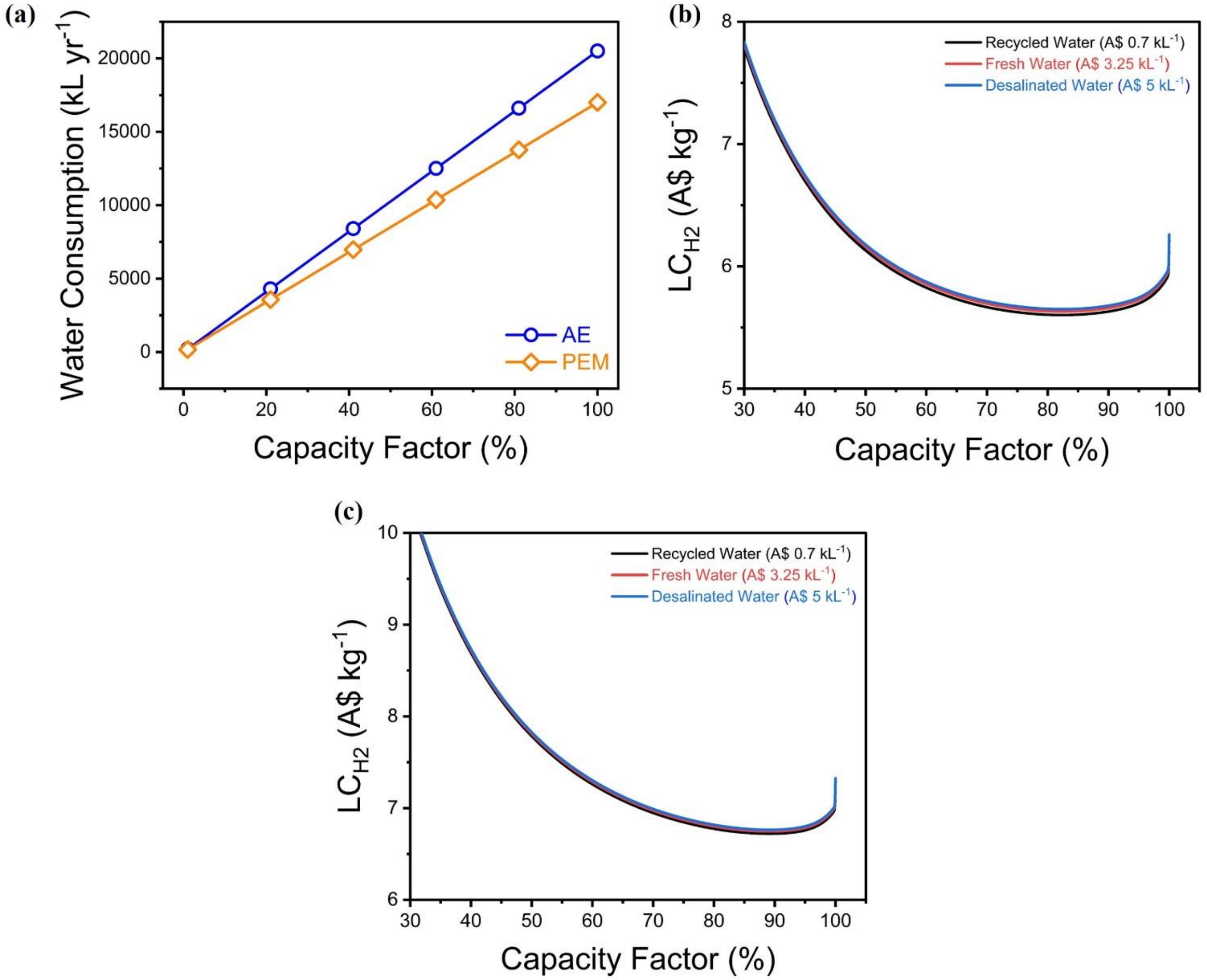

Figure S3. (a) Water consumption rates of the electrolyzer system (AE and PEM). The influence of water pricing on LC_{H2} for 10 MW (b) AE and (c) PEM system. The AE system requires 1.5 L per Nm^3 of hydrogen generated (11.2 L kg^{-1}), while the PEM system has a lower water requirement 1.0 L Nm^{-3} of H_2 produced (10 L kg^{-1}). To determine the influence of water pricing on LC_{H2}, we assumed the grid scenario as the base case as the capacity factor for this configuration is fixed. The water pricing was adopted from literature(Shwisher *et al.*, 2019), which suggested that recycled water, freshwater and desalinated water will cost A\$0.7 kL^{-1}, A\$3.25 kL^{-1} and A\$5 kL^{-1}, respectively. From these results, it is clear that water consumption has a very low impact on the overall LC_{H2}. However, it is essential to note that this pricing is based on wholesale market pricing, in case water cannot be sourced from a retailer, and a self-sustained water supply would have to be considered. In such a scenario, the installed water plant's capital and operating cost would add to the cost of the facility and influence LC_{H2}. This scenario was not considered in this analysis but would be an important consideration during a detailed design of actual electrolyzer projects. **Related to Table 1, main text and STAR Methods.**

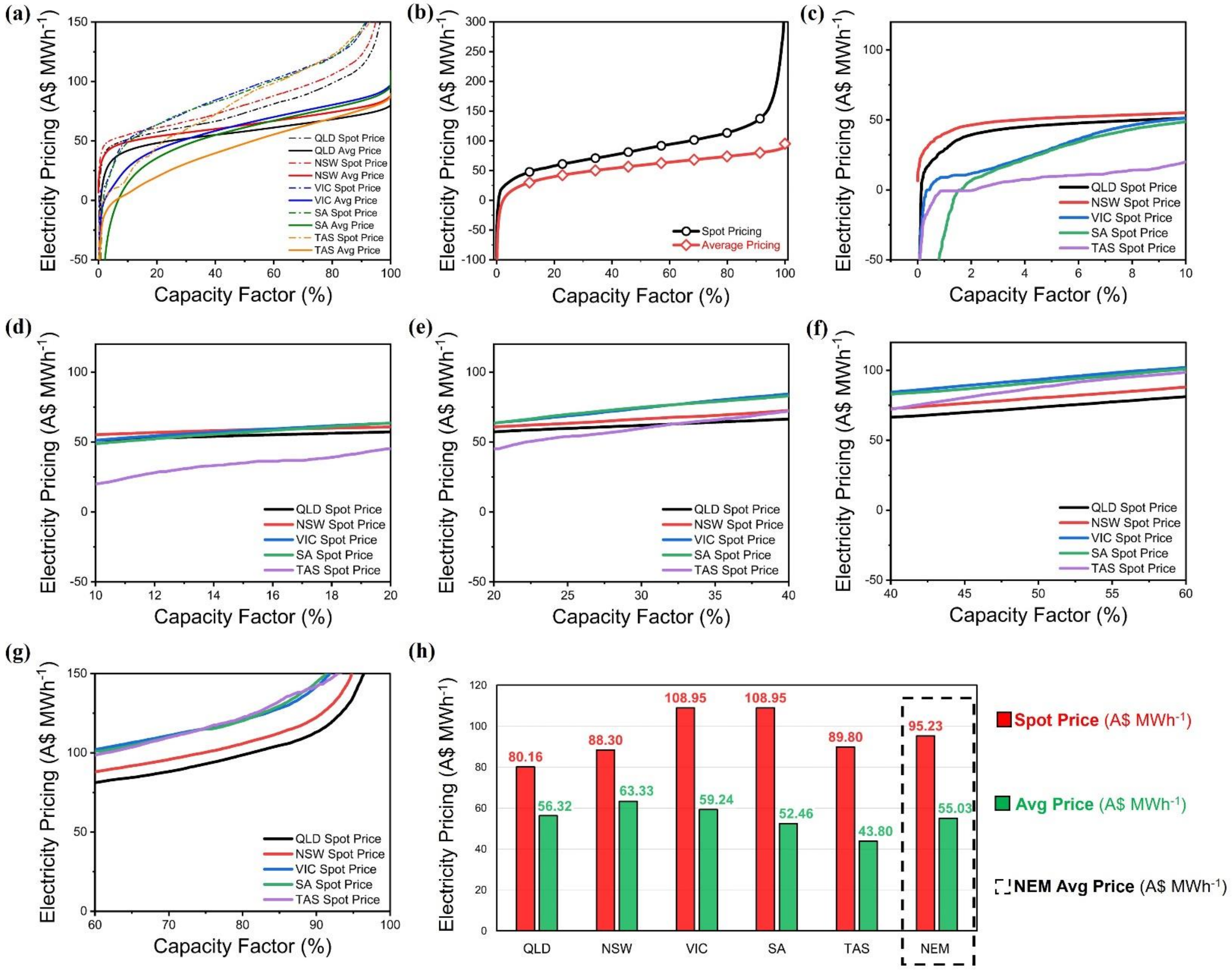

Figure S4. The outlook of the wholesale electricity pricing reported for the FY2018-19 for the Australian National Energy Market (data provided by the Australian Energy Market Operator – AEMO). (**a**) The electricity price varies between states and across the capacity factor of the grid due to the variation in spot and average wholesale pricing. Given the spot price varies depending on the market, the average price, i.e., the cumulative spot pricing concerning time can provide more simplistic guidance on how flexible loads can operate within a volatile market. Furthermore, each states' spot and average prices can then be aggregated to represent the (**b**) outlook of the wholesale pricing for the NEM. However, it is still essential to analyze the volatility of the spot price, as high and very low electricity pricing will significantly impact the average pricing. To simplify the price duration curves, we have truncated the pricing over (**c-g**) different grid capacity factors. It is observed that there is high volatility (predominantly negative pricing) at low-capacity factors (<20%). Afterwards, the market in each state shows a steady increase (up until 70% capacity factor) and high volatility (high electricity pricing) is again observed at very high-capacity factors (>80%). The overall comparison of the (**h**) pricing for the states shows that Tasmania (TAS), South Australia (SA), and Queensland (QLD) had the lowest electricity pricing as compared to New South Wales (NSW) and Victoria (VIC) that had the higher end electricity pricing. These overall costs were calculated as a total arithmetic mean of the spot and average price curves for each state, while the overall cost of the NEM was represented by taking the arithmetic average of the cumulative spot and average prices of all the states. **Related to Figure 3.**

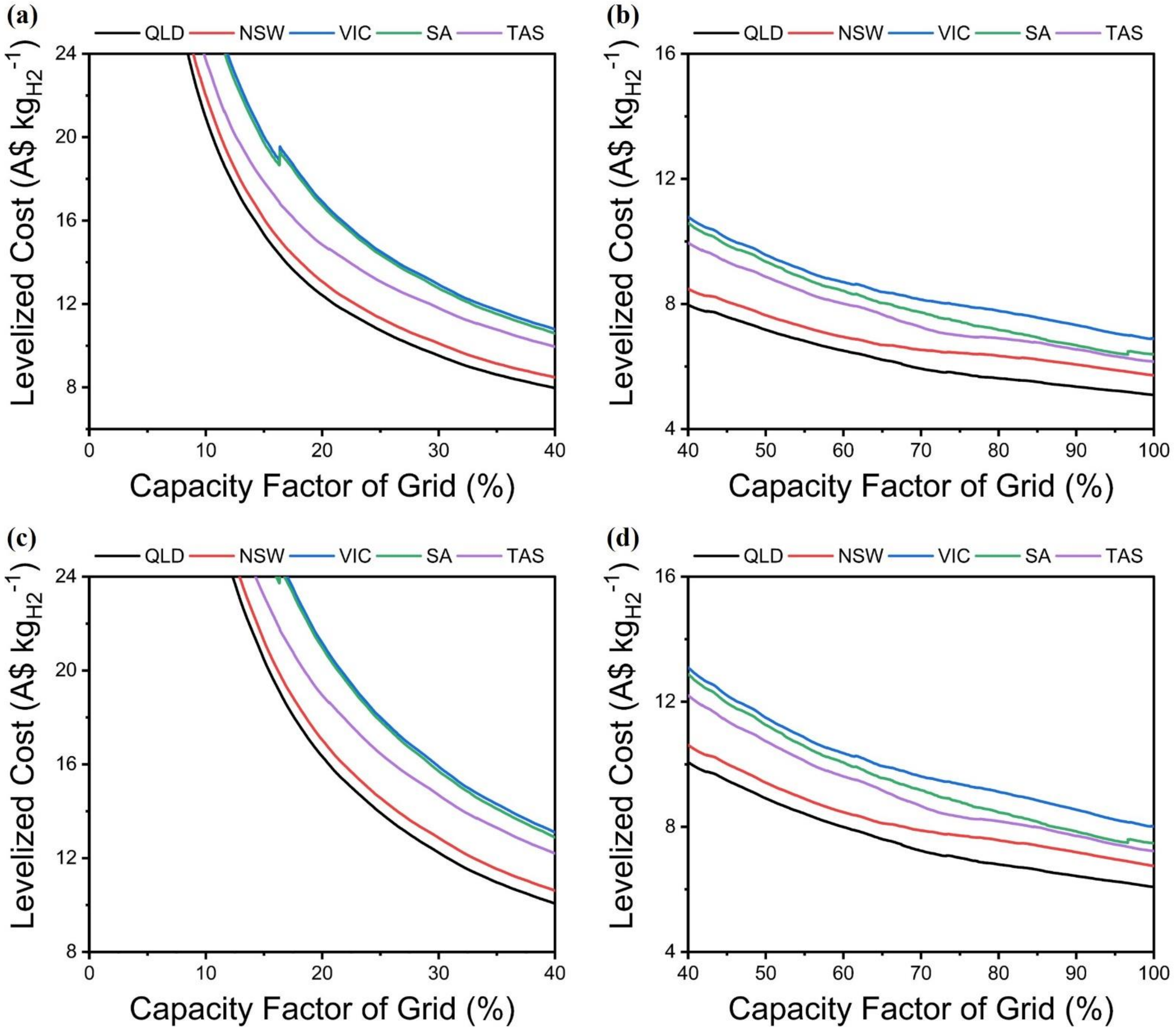

Figure S5. The levelized cost distribution for the grid operated (**a,b**) AE and (**c,d**) PEM system as a function of the capacity factor. The levelized cost has a reversed trend compared to electricity pricing. With the increase of capacity factor, the electrolyzer generates larger amounts of hydrogen that offset the increased operating costs (due to subsequent increase in average electricity pricing – Figure **S11**)—reaffirming that a tradeoff exists between a higher electricity cost and the benefit of lower cost hydrogen. Provided that the electricity pricing is for the whole year, the average of this price duration would represent the overall LC_{H2} of hydrogen generated. **Related to Figure 3.**

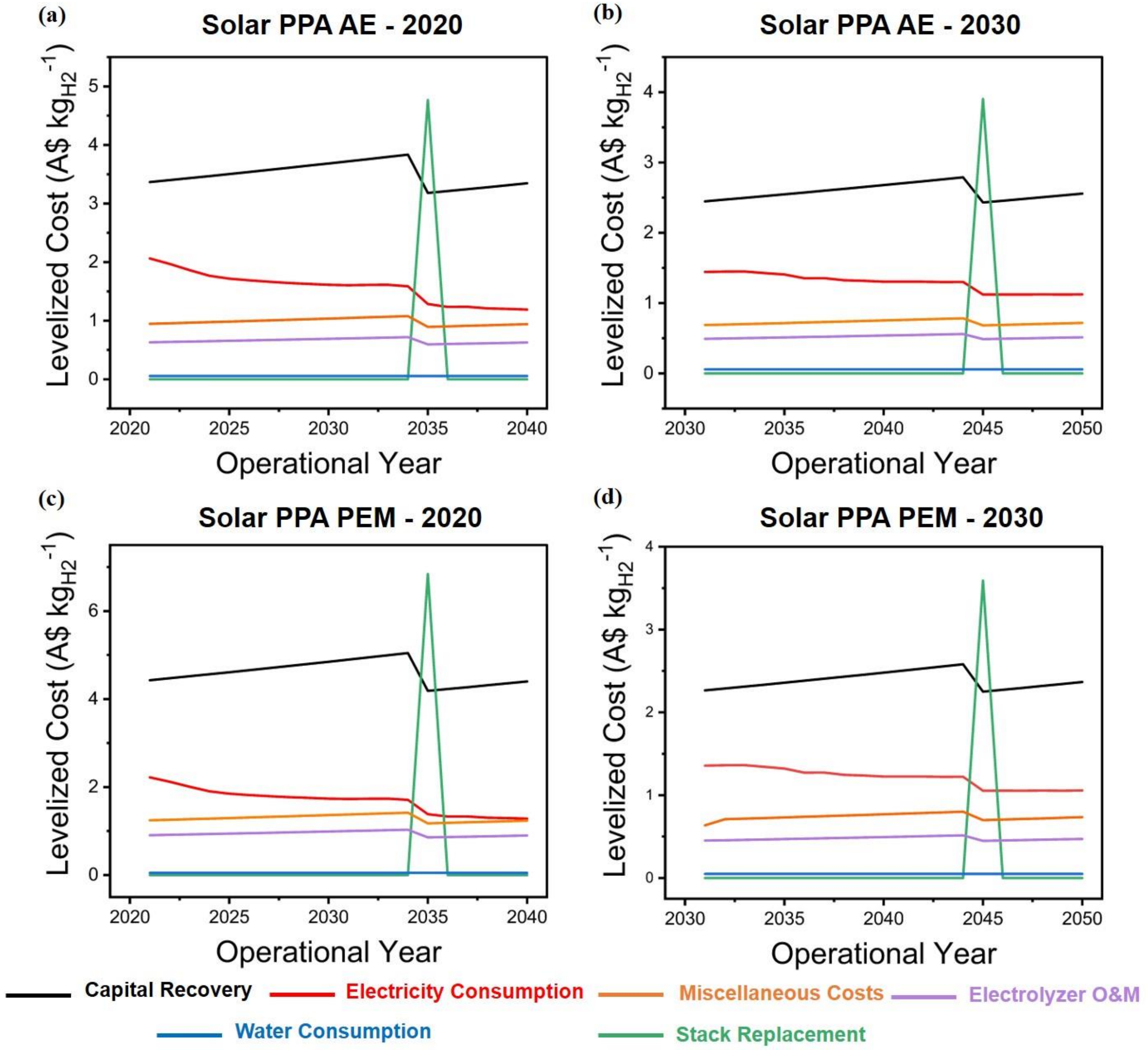

Figure S6. The yearly component breakdown of levelized cost of hydrogen (over 20-year project life) of solar PPA powered AE system operated in **(a)** Window 1: 2021 – 2040 and (**b**) Window 2: 2031 – 2050; and for solar PPA powered PEM system operated in in (**c)** Window 1: 2021 – 2040 and (**d**) Window 2: 2031 – 2050. In both cases of technology and operational period, the actual cost of the capital recovery and operating costs (except for electricity consumption) remains the same but have an increasing share in the LC_{H2} due to lesser amount of hydrogen produced each subsequent year due to electrolyzer degradation. On the other hand, electricity cost has a decreasing trend due to improved cost of PPA in each year. Stack replacement is expected to be due in 2035 (40,000 hours lifetime, 31% capacity factor = 14 years), leading to increased operating expenditure. However, stack replacement has an advantage as more efficient stacks would be available post-2030 compared to 2020. Thus, the stack will generate a higher amount of hydrogen compared to before, causing the curves to experience a step-down, as the operating and capital recovery costs are levelized over a larger amount of produced hydrogen. Afterwards, the curves retake an upward trend as the electrolyzer system degrades. **Related to Figure 4.**

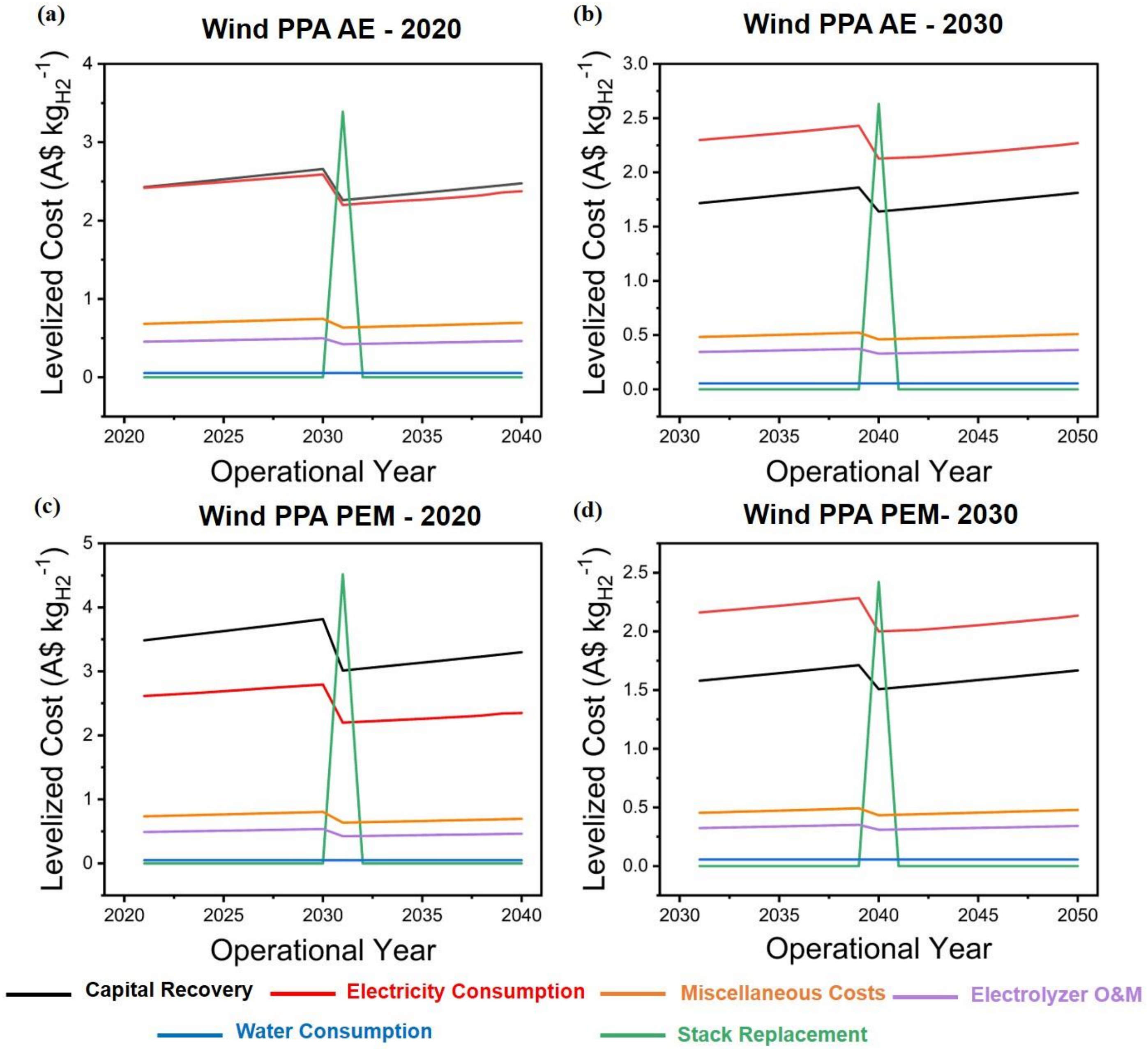

Figure S7. The yearly component breakdown of levelized cost of hydrogen (over 20-year project life) of wind PPA powered AE system operated in **(a)** Window 1: 2021 – 2040 and (**b**) Window 2: 2031 – 2050; and for wind PPA powered PEM system operated in (**c)** Window 1: 2021 – 2040 and (**d**) Window 2: 2031 – 2050. In In both cases of technology and operational period, the actual cost of the capital recovery and operating costs (except for electricity consumption) remains the same but have an increasing share in the LC_{H2} due to lesser amount of hydrogen produced each subsequent year due to electrolyzer degradation. On the other hand, electricity cost has a decreasing trend due to improved cost of PPA in each year. Stack replacement is expected to be due in 2035 (40,000 hours lifetime, 31% capacity factor = 14 years), leading to increased operating expenditure. However, stack replacement has an advantage as more efficient stacks would be available post-2030 compared to 2020. Thus, the stack will generate a higher amount of hydrogen than before, causing the curves to experience a step-down, as the operating and capital recovery costs are levelized over a larger amount of produced hydrogen. Afterwards, the curves retake an upward trend as the electrolyzer system degrades. **Related to Figure 4.**

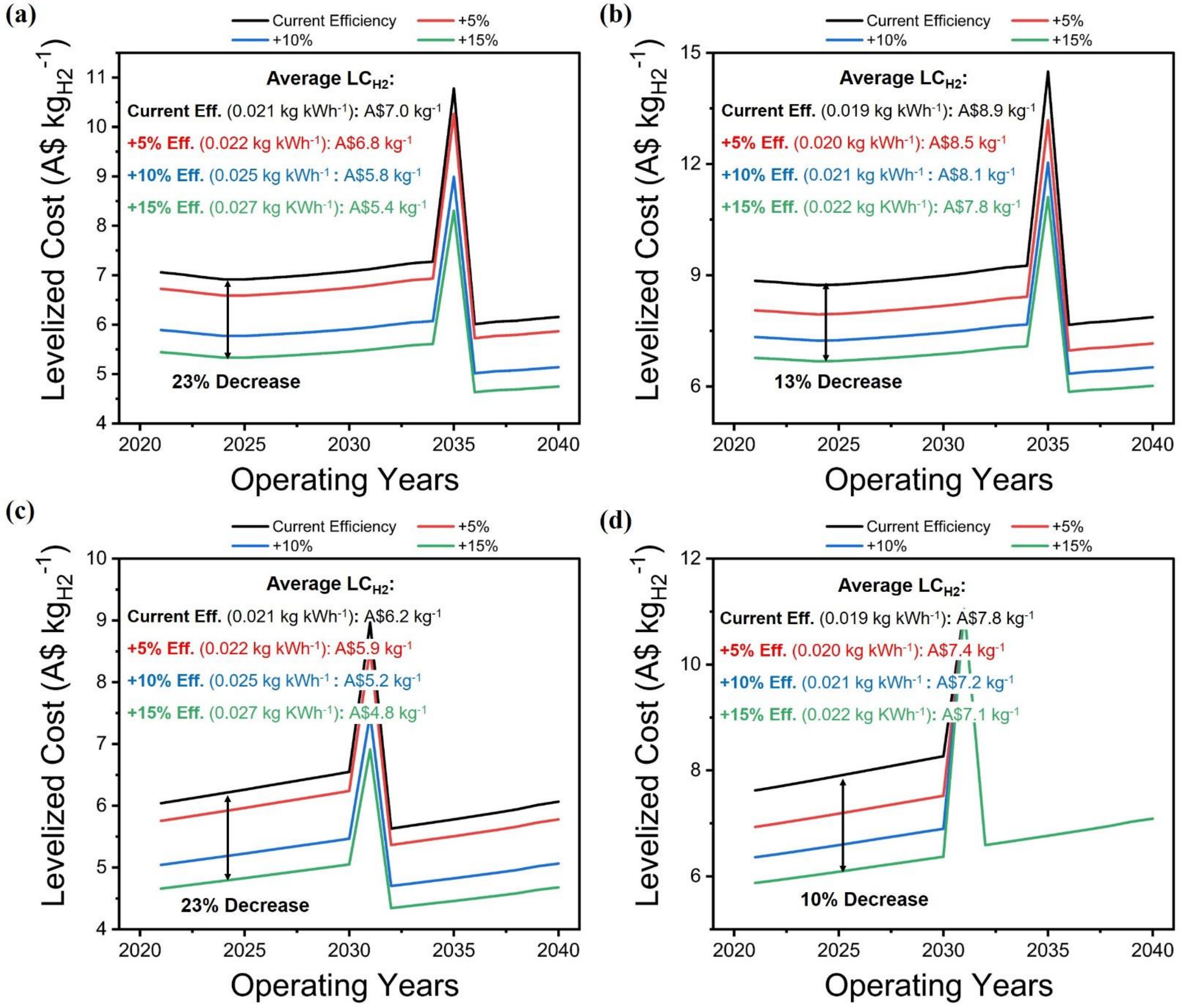

Figure S8. The influence of improving the electrolyzer efficiency on the LC_{H2} of the solar PPA powered (**a**) AE and (**b**) PEM systems and the wind PPA powered (**c**) AE and (**d**) PEM system. The average LC_{H2} can be decreased by ~ 23% for AE and between 10 to 13% for PEM systems if the electrolyzer system efficiency can be increased by 15% of current levels. The percentage decrease represented in the figure was calculated as the ratio of the difference between the LC_{H2} at the base case current efficiency (shown in black) and the LC_{H2} at each considered improved efficiency level (red, blue and green) to the base case LC_{H2}. **Referred to in the main text**, **related to Figure 4.**

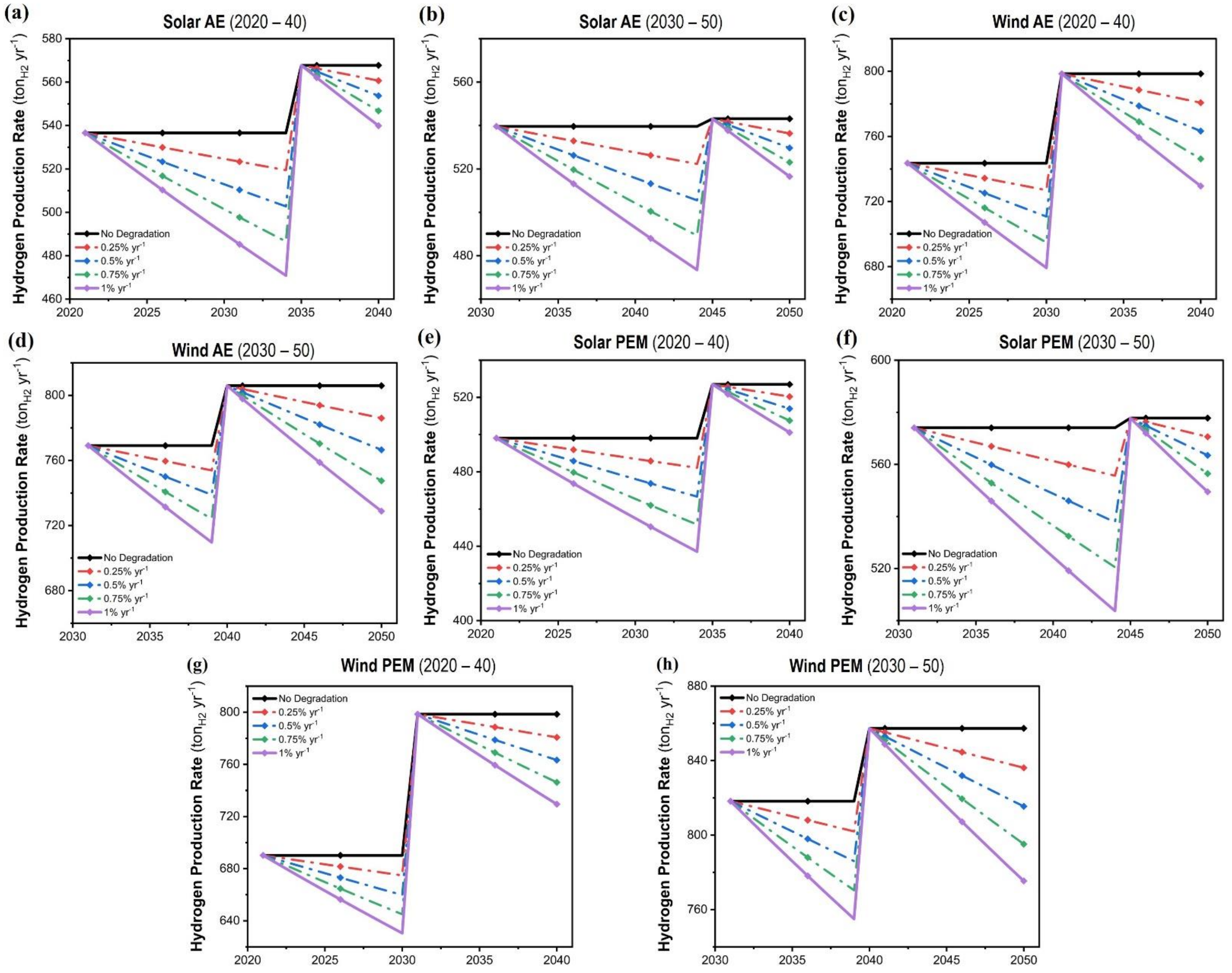

Figure S9. Degradation profile of the (**a-d**) AE and (**e-h**) PEM electrolyzers while operating with the Solar and Wind PPA powered systems, respectively, as a function of different assumed degradation rates. The spike in the production rate is observed after stack replacement, after which the new electrolyzer stack is assumed to degrade again at the considered degradation rates. The production rates for window 2 operation (2030 – 2050) is higher due to more efficient electrolyzers expected to be available by 2030 compared to today (2020). Wind operated systems have a higher production rate than the solar-powered systems due to higher capacity factors. **Referred to in the main text**, **related to Figure 4.**

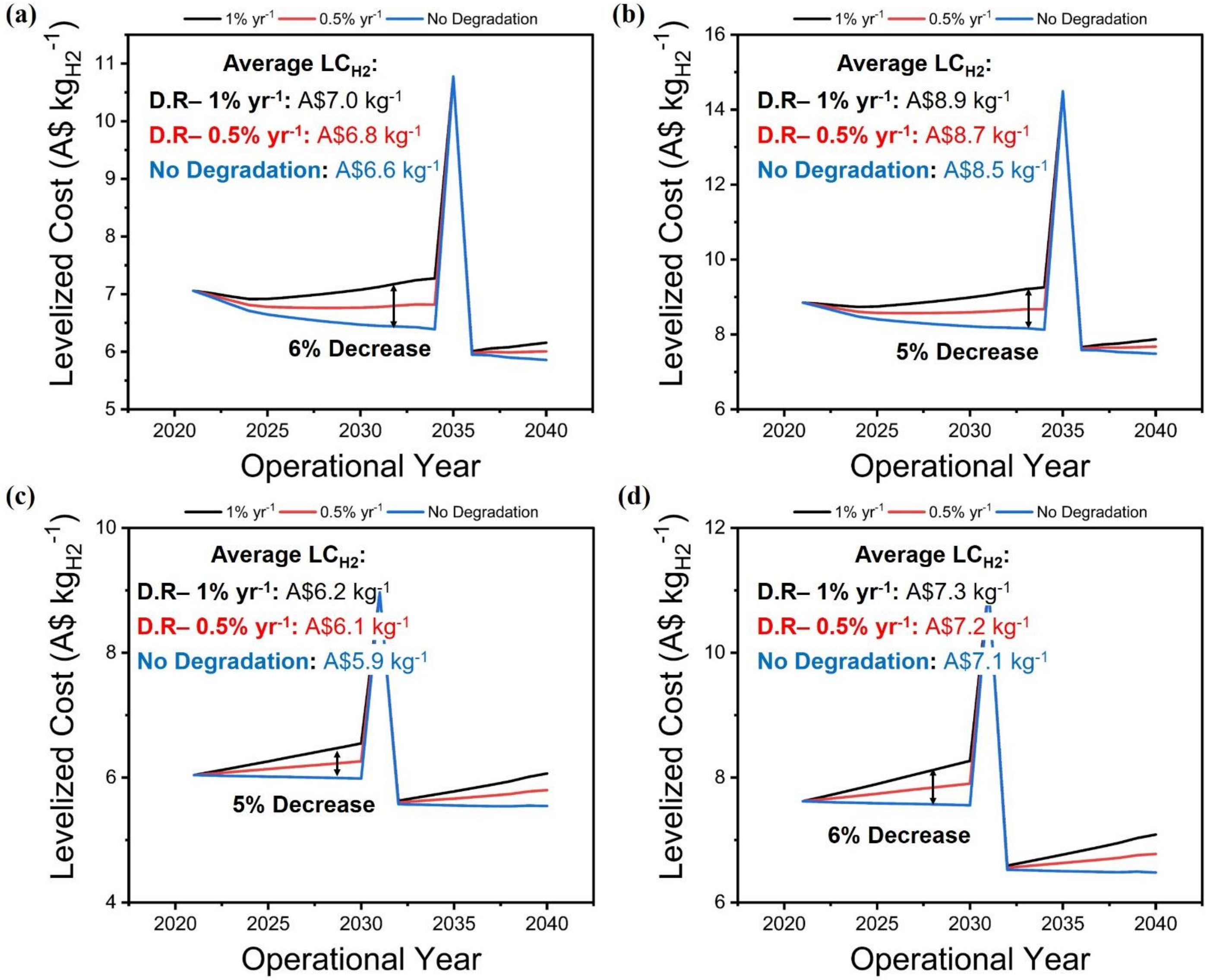

Figure S10. The influence of reducing the electrolyzer degradation rate on the LC_{H2} of the Solar PPA powered (**a**) AE and (**b**) PEM systems and the Wind PPA powered (**c**) AE and (**d**) PEM system. Improving the degradation rate does reduce the LC_{H2} but not as significantly as improving efficiency. The percentage decrease represented in the figure was calculated as the ratio of the difference between the LC_{H2} at the base case degradation rate (shown in black) and the LC_{H2} at each considered improved degradation rate (red and blue) to the base case LC_{H2}. **Referred to in the main text**, **related to Figure 4.**

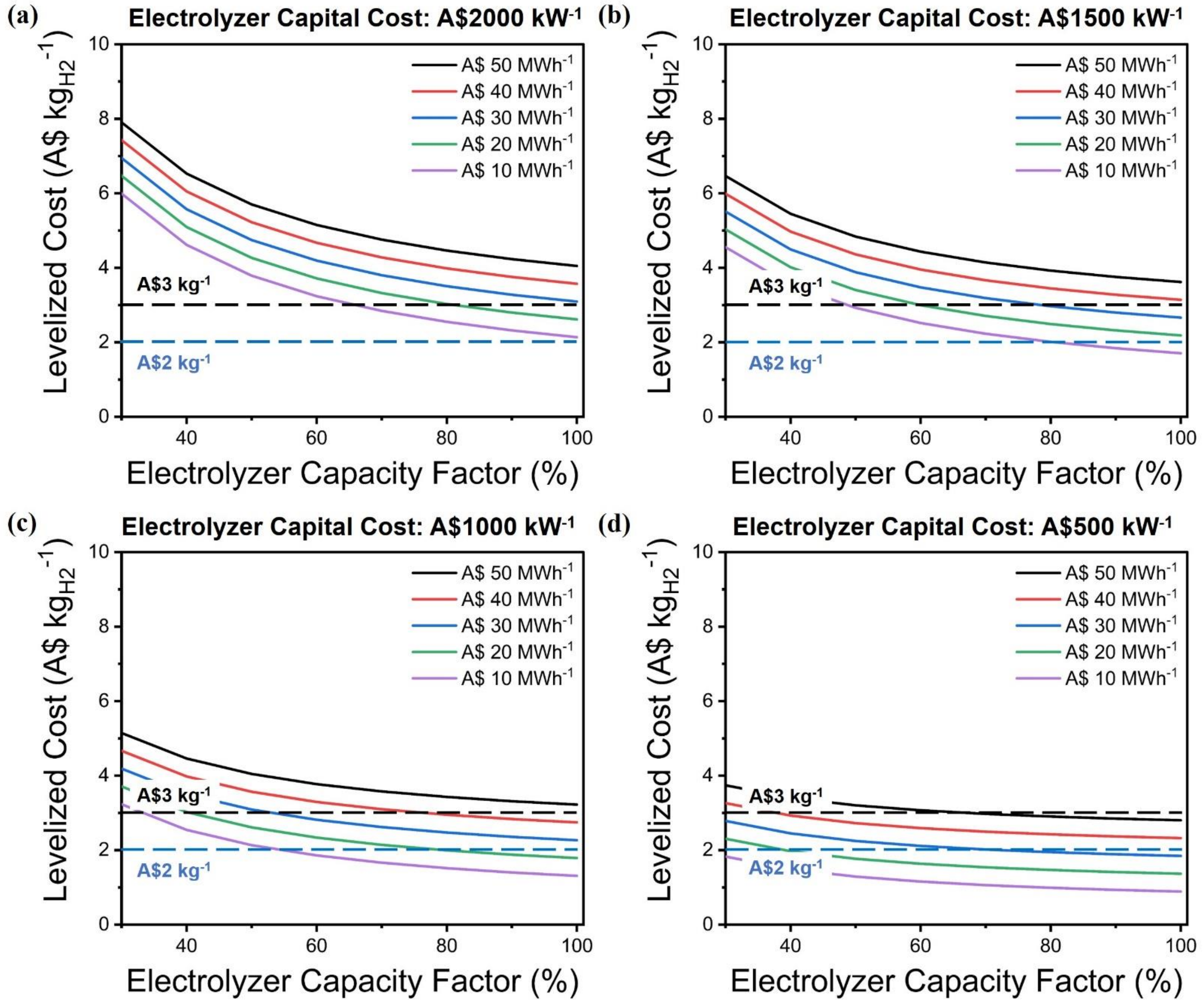

Figure S11. LC_{H2} of the electrolyzer system (SEC: 0.02 kg kWh^{-1}) represented as a function of decreasing (**a-d**) electrolyzer capital cost (including direct and indirect costs) and various renewable electricity pricing and availability (capacity factor). The results reveal that achieving LC_{H2} below A$3 kg^{-1} would require electrolyzer CAPEX $\leq$ A$1000 kW^{-1} and electricity costs <A$40 MWh^{-1} for a capacity factor of 70%. Similarly, to reach LC_{H2} <A$2 kg^{-1} would require electrolyzer CAPEX < A$1000 kW^{-1} and electricity costs <A$20 MWh^{-1} for a capacity factor of 80%. Whereas for electrolyzer CAPEX $\leq$ A$500 kW^{-1}, LC_{H2} can be reduced to A$3 kg^{-1} at an electricity price <A$50 MWh^{-1} (capacity factor: 60%) and to A$2 kg^{-1} at electricity price <A$30 MWh^{-1} (capacity factor:60%). The black and blue lines were added as a reference to compare the achieved costs against the A$3 kg^{-1} (black dashed line) and A$2 kg^{-1} (blue dashed line) targets. **Referred to in main text, related to Figure 5 and 6.**

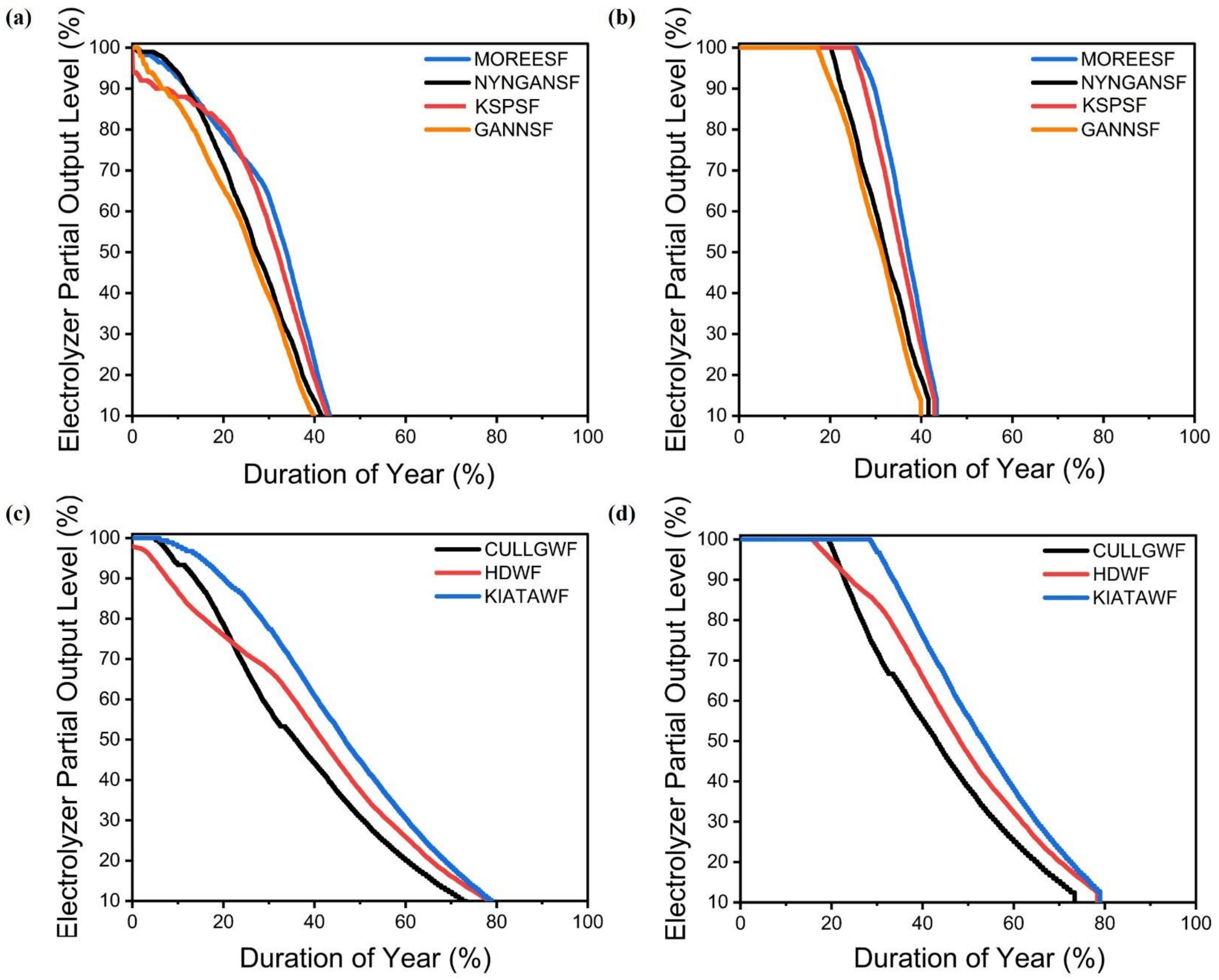

Figure S12. The electrolyzer partial output level represented as a distribution across an operational year while operating through (a) an equivalent (10 MW) solar PV plant (b) 10 MW wind, (c) oversized 14 MW solar PV and (d) oversized 12.5 MW wind farm. The electrolyzer partial output level (y-axis) is the ratio of the actual output (nominal capacity) to the nameplate capacity (10 MW). While the duration of the year (x-axis) represents the fraction of the total 8,760 hours in a year. These curves were drawn based on the actual generation data – duration curves (30 min. intervals) for the considered Australian Solar and Wind Farms. These include the Moore (MOREESF) and Nyngan (NYNGAN) Solar Farm in New South Wales as well the Kidston Solar Project (KSP) in Queensland and Gannawarra Solar Farm (GANNSF) in Victoria. Similarly, the Cullerin Range Wind Farm (CULLRGWF), Hornsdale Wind Farm (HDWF) in South Australia and Kiata Wind Farm (KIATAWF) in the Australian Capital Territory. **Related to STAR Methods.**

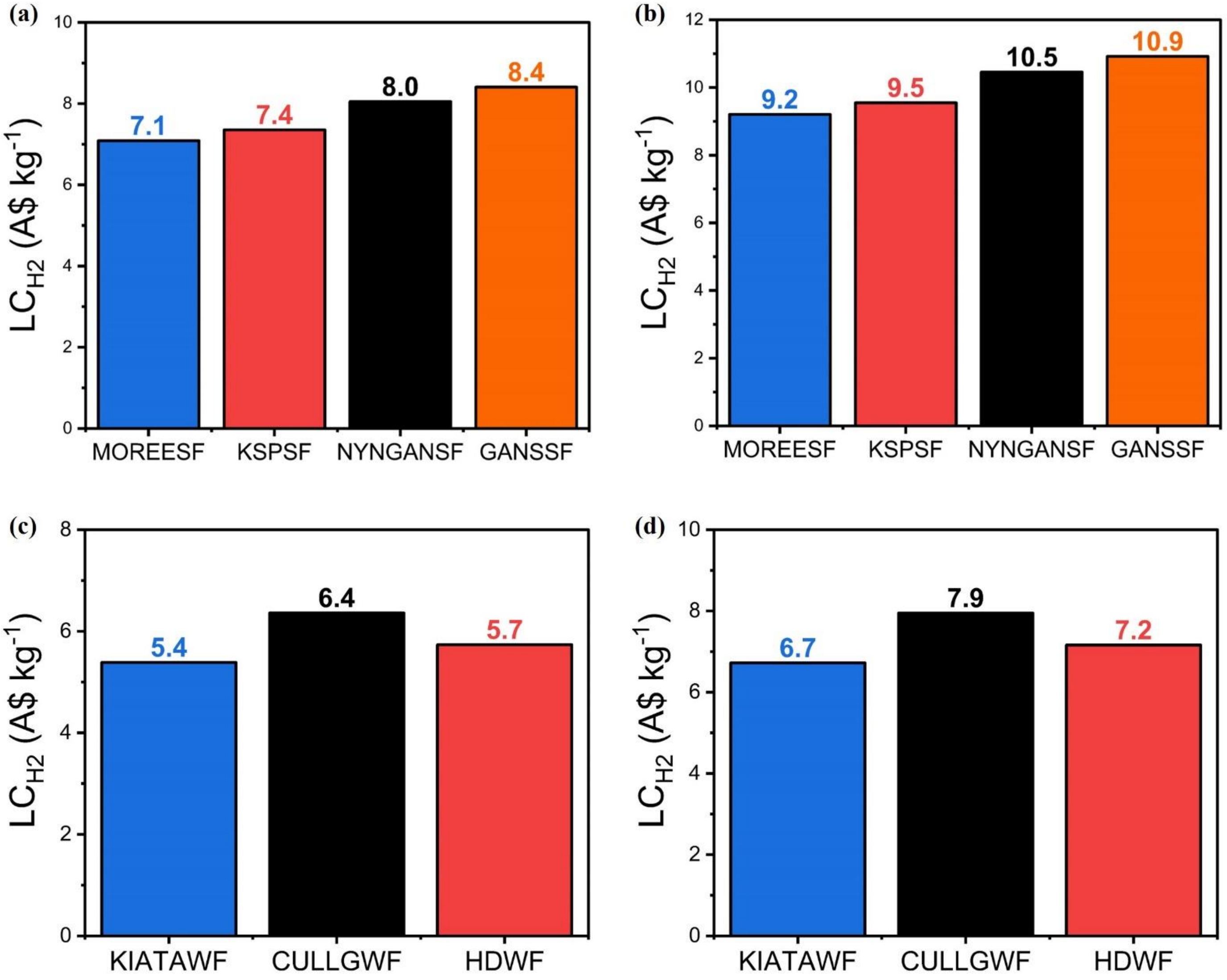

Figure S13. Estimated average LC_{H2} for off-grid 10 MW **(a)** AE and **(b)** PEM system integrated with oversized 15 MW solar farms. Note that MOREESF, KSPF, NYNGANSF and GANSSF represent different solar PV farms around Australia. Estimated average LC_{H2} for off-grid 10 MW **(c)** AE and **(d)** PEM system integrated with oversized 15 MW wind farms. Note that KIATAWF, CULLGWF and HDWF are representative wind farms around Australia. **Referred to in the main text**, **related to Figure 7.**
